# MRF4 negatively regulates adult skeletal muscle growth by repressing MEF2 activity

**DOI:** 10.1038/ncomms12397

**Published:** 2016-08-03

**Authors:** Irene Moretti, Stefano Ciciliot, Kenneth A. Dyar, Reimar Abraham, Marta Murgia, Lisa Agatea, Takayuki Akimoto, Silvio Bicciato, Mattia Forcato, Philippe Pierre, N. Henriette Uhlenhaut, Peter W. J. Rigby, Jaime J. Carvajal, Bert Blaauw, Elisa Calabria, Stefano Schiaffino

**Affiliations:** 1 Venetian Institute of Molecular Medicine (VIMM), via Orus 2, 35129 Padova, Italy; 2 Molecular Endocrinology, Institute for Diabetes and Obesity, Helmholtz Zentrum München, Business Campus Garching, Parkring 13, D-85748 Garching, Germany; 3 Department of Biomedical Sciences, University of Padova, via Ugo Bassi 58/B, 35131 Padova, Italy; 4 Center for Genome Research, Department of Life Sciences, University of Modena and Reggio Emilia, Via Campi 287, 41125 Modena, Italy; 5 Centre d'Immunologie de Marseille-Luminy, Aix-Marseille Université, INSERM, CNRS, 13288, Marseille, France; 6 Institute for Research in Biomedicine (iBiMED), and Aveiro Health Sciences Program, University of Aveiro, 3810-193 Aveiro, Portugal; 7 Division of Cancer Biology, The Institute of Cancer Research, Chester Beatty Laboratories, 237, Fulham Road, London SW3 61B, UK; 8 Molecular Embryology Team, Centro Andaluz de Biología del Desarrollo, CSIC-UPO-JA, Carretera de Utrera Km1, 41013 Seville, Spain

## Abstract

The myogenic regulatory factor MRF4 is highly expressed in adult skeletal muscle but its function is unknown. Here we show that *Mrf4* knockdown in adult muscle induces hypertrophy and prevents denervation-induced atrophy. This effect is accompanied by increased protein synthesis and widespread activation of muscle-specific genes, many of which are targets of MEF2 transcription factors. MEF2-dependent genes represent the top-ranking gene set enriched after *Mrf4* RNAi and a MEF2 reporter is inhibited by co-transfected MRF4 and activated by *Mrf4* RNAi. The *Mrf4* RNAi-dependent increase in fibre size is prevented by dominant negative MEF2, while constitutively active MEF2 is able to induce myofibre hypertrophy. The nuclear localization of the MEF2 corepressor HDAC4 is impaired by *Mrf4* knockdown, suggesting that MRF4 acts by stabilizing a repressor complex that controls MEF2 activity. These findings open new perspectives in the search for therapeutic targets to prevent muscle wasting, in particular sarcopenia and cachexia.

## Abstract (web summary)

Mrf4 is a transcription factor important for muscle development, but despite high expression its function in adults is unknown. Here the authors show that interfering with Mrf4 in adult mice leads to muscle hypertrophy by activating MEF2-dependent transcription and promoting protein synthesis.

## Graphical abstract

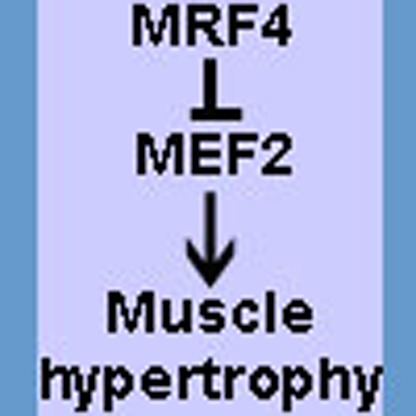

The basic helix-loop-helix (bHLH) family of myogenic regulatory factors (MRFs) comprises four members, MyoD, myogenin, myogenic factor 5 (Myf5) and MRF4, which play key roles in skeletal muscle commitment and differentiation^1^. The *MyoD* and *Myf5* genes are involved in muscle commitment during embryogenesis, whereas myogenin has a crucial downstream role in the differentiation of committed muscle progenitors into myofibres. *Mrf4* differs from the other family members in that it has a biphasic pattern of expression during mouse development^2^. *Mrf4* is transiently expressed at the same time as *Myf5* at the onset of myogenesis in the embryo^3^ and can function as a determination gene, as some myogenesis takes place in a double *Myf5/MyoD* mutant in which *Mrf4* is not compromised^4^. A later phase of *Mrf4* expression starts during fetal development and continues throughout postnatal stages and is by far the predominant MRF expressed in adult muscle fibres^5^. However, the function of MRF4 in adult muscle is not known.

We sought to understand the role of MRF4 in adult skeletal muscle using an RNA interference (RNAi) approach. Here we show that *Mrf4* knockdown in adult skeletal muscle causes a striking increase in muscle fibre size, suggesting that MRF4 is a negative regulator of muscle growth. Muscle hypertrophy induced by *Mrf4* RNAi is accompanied by increased expression of muscle-specific genes, including those encoding proteins involved in the sarcomere, the membrane cytoskeleton, the excitation–contraction coupling apparatus and energy metabolism. This effect is dependent on an increase in MEF2 transcriptional activity and the consequent upregulation of MEF2 target genes. We show that the hypertrophic effect of *Mrf4* RNAi is abolished by dominant negative MEF2, while myofibre hypertrophy is induced by constitutively active MEF2. The identification of two transcription factors that act together to regulate growth in adult muscle raises interesting possibilities for the treatment of muscle wasting conditions.

## Results

### *Mrf4* RNAi induces adult muscle growth and protein synthesis

Short hairpin RNA (shRNA) sequences targeting *Mrf4* mRNA were inserted into pSUPER plasmids and co-transfected in to cultured HEK-293 cells together with a plasmid encoding myc-tagged rat MRF4. A vector containing shRNA sequences targeting *LacZ* was used as a negative control. Two *Mrf4*-specific shRNAs, referred to as M1 and M2, were found to markedly decrease the expression of MRF4 (Supplementary Fig. 1a) and were thus selected for *in vivo* studies. Plasmids coding for M1 and M2 were then electroporated in to rat muscles, together with a plasmid encoding GFP. A marked decrease in nuclear staining for the endogenous MRF4 was seen in transfected muscle fibres, identified by GFP expression, compared with untransfected fibres within the same muscles (Supplementary Fig. 1b). Unlike MyoD and myogenin, which are prevalent in fast or slow muscles, respectively, we found that MRF4 is expressed at similar RNA and protein levels in the fast extensor digitorum longus (EDL) and slow soleus (SOL) muscles (not shown), in agreement with previous studies^6^,^7^. Therefore, we examined the effect of M1 and M2 in both EDL and SOL muscles.

**Figure 1:**
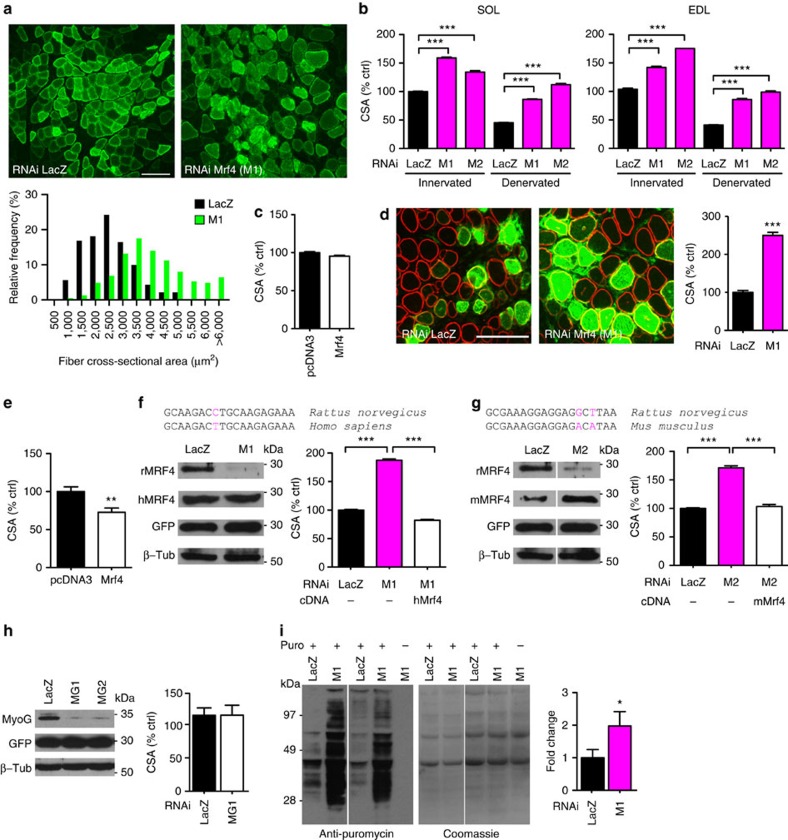
*Mrf4* RNAi induces myofibre hypertrophy and protein synthesis in adult muscles. (**a**) Rat soleus muscles co-transfected with GFP and *LacZ or Mrf4* shRNAs and examined 14 days later. Scale bar, 100 μm. Cross-sectional area (CSA) of transfected fibres is shown below. Two hundred GFP-positive fibres from each muscle were analysed. (**b**) CSA of muscle fibres in innervated or 7 d denervated SOL and EDL muscles transfected with two different *Mrf4* shRNAs (M1 and M2). Values normalized to fibres transfected with *LacZ* shRNAs in innervated muscles. Measures on 58 muscles, about 5 muscles per group, 15,485 fibres. (**c**) Mrf4 overexpression in adult SOL. CSA of muscle fibres transfected with Mrf4 cDNA normalized to control muscles (*n*=3). (**d**) Fibre size is increased in regenerating SOL co-transfected at day 3 after injury with GFP and *LacZ or Mrf4* shRNAs, and examined 7 days later. Scale bar 100 μm. Right: CSA of regenerating fibres transfected with *Mrf4* shRNAs (M1) normalized to control (*n*=3). (**e**) Fibre size is reduced by Mrf4 overexpression in regenerating muscle (*n*=3). (**f**) Rescue experiment for M1. The sequence recognized by M1 shRNA in rat Mrf4 transcripts (rMrf4) has a single base difference in human Mrf4 (hMrf4). hMrf4 is not silenced by M1 in HEK-293 cells (left), as shown by western blotting with anti-MRF4. Fibre growth induced by M1 in 14 days denervated SOL is prevented by M1-resistant hMrf4 (right, *n*=5). (**g**) Rescue experiment for M2. Same as in f, but using M2 shRNA specific for rat Mrf4 and M2-resistant mouse Mrf4 (mMrf4) (*n*=5). (**h**) Fibre size is not affected by *myogenin* knockdown. Left: HEK-293 cells transfected with myogenin cDNA (MyoG) and co-transfected with two shRNAs targeting myogenin (MG1 and MG2). GFP was co-transfected to determine transfection efficiency. Right: fibre size in unchanged by MG1 (*n*=3). (**i**) Increased Protein synthesis is increased in SOL by *Mrf4* shRNAs (M1), as revealed by puromycin incorporation and immunostaining for puromycin-labelled peptides. Quantification is on the right (*n*=4). Data are presented as mean±s.e.m. from at least three independent experiments. Statistical analysis with Student's two-tailed *t*-test (*P<0.05, **P<0.01, ***P<0.001).

The most obvious change induced by MRF4 knockdown was the marked hypertrophy of most transfected fibres compared with *LacZ* shRNA controls (Fig. 1a,b) and to non-transfected fibres in the same muscle (Fig. 1a and Supplementary Fig. 2). Muscle fibre hypertrophy was evident at 7 and 14 days post transfection in both innervated and denervated muscles, denervation atrophy being prevented by *Mrf4* RNAi (Fig. 1b and Supplementary Fig. 3). In contrast, muscle fibre size was unaffected by overexpression of *Mrf4* in adult muscles (Fig. 1c). We also examined the effects of *Mrf4* knockdown and overexpression in regenerating muscles. Regenerating muscle growth was strikingly accelerated by *Mrf4* knockdown, with fibre size more than doubled compared with controls (Fig. 1d and Supplementary Fig. 4). A smaller but significant change in the opposite direction was induced by *Mrf4* overexpression in regenerating muscle, with fibre size being reduced by about 20% compared with control (Fig. 1e).

To validate the specificity of our RNAi experiments and rule out the possibility that the observed changes were due to off-target effects, we performed rescue experiments with RNAi-resistant target genes. The sequence recognized by M1 shRNA in rat *Mrf4* has a single base difference in human *Mrf4*, so that expression of the human gene is not silenced by M1 in cultured HEK-293 cells (Fig. 1f). *In vivo* transfection experiments showed that the increase in fibre size induced by *Mrf4* RNAi was completely abrogated when a plasmid encoding human *Mrf4* was co-transfected with M1 (Fig. 1f). A similar rescue experiment with identical results was performed with M2, using mouse *Mrf4*, which is M2-resistant because of a two-base difference in the sequence recognized by M2 shRNA (Fig. 1g). Next, we asked whether the effect of *Mrf4* knockdown on muscle growth is specific for this member of the MRF family and tested the effect of shRNAs targeting *myogenin*. However, no effect on muscle fibre size was observed when myogenin-specific shRNAs were transfected in to adult skeletal muscle (Fig. 1h), in agreement with the finding that muscle weight was unchanged by deletion of the myogenin gene in adult innervated muscles using an inducible knockout model^8^.

Muscle hypertrophy is always accompanied by increased protein synthesis^9^. To monitor protein synthesis in transfected muscles, we used a procedure based on the incorporation of puromycin into nascent peptides^10^. As shown in Fig. 1i, *Mrf4* RNAi induced a significant increase in the amount of puromycin-labelled peptides compared with *LacZ* RNAi controls. This finding shows that protein synthesis is markedly increased during muscle hypertrophy induced by loss of MRF4, as in other models of muscle hypertrophy.

### Muscle-specific genes are upregulated by *Mrf4* knockdown

**Figure 2:**
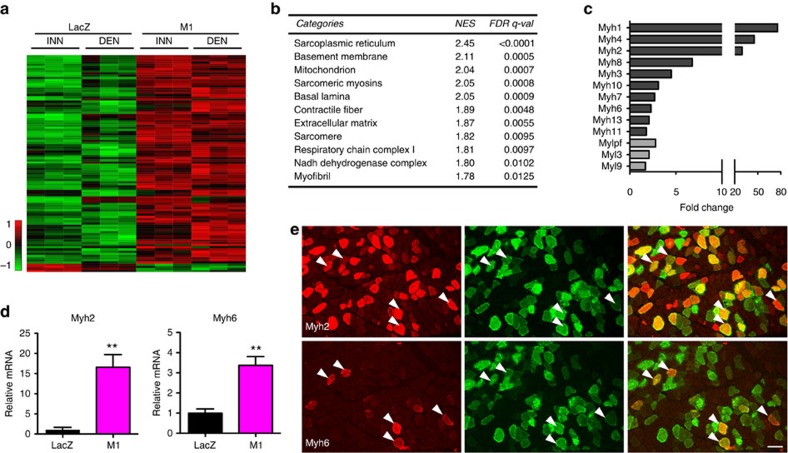
Differentially expressed genes in muscles transfected with Mrf4 shRNAs. (**a**) Heatmap representing the top-ranking 100 genes showing differential expression between M1 and control. Each lane corresponds to one animal, three animals per group were examined. Note that innervated (INN) and denervated (DEN) M1 groups display similar changes in gene expression and that genes upregulated by M1 are predominant in this heatmap. (**b**) Gene Set Enrichment Analysis (GSEA) reporting the groups of genes of the GO Cellular Component positively enriched in muscles transfected with shRNAs against *Mrf4* (M1 sequence). NES: Normalized Enrichment Score; FDR: false discovery rate. (**c**) Myosin heavy and light chains upregulated by *Mrf4* knockdown (*P*<0.05; fold change≥1.5). (**d**) Increased levels of myosin heavy chain (MyHC) transcripts coded by *Myh2* and *Myh6* in muscles transfected with *Mrf4* RNAi (M1) compared to control *LacZ* RNAi, as determined by qPCR (*n*=3, same samples used for microarray analysis in **a**). Data are presented as means±s.e.m. Statistical analysis was performed using Student's two-tailed *t*-test, **P<0.01. (**e**) SOL muscle transfected with *Mrf4* shRNA and examined at 14 days after transfection. Serial sections stained with anti-MyHC-2A (*Myh2*) and MyHC-α cardiac (*Myh6*) reveal that a number of transfected fibres staining for MyHC-2A are also reactive for MyHC-α cardiac. Scale bar, 100 μm.

To address the mechanism of muscle hypertrophy induced by *Mrf4* knockdown, we performed microarray analysis of innervated and denervated SOL muscles transfected with shRNA to *Mrf4* (M1 sequence) and compared them with muscles transfected with shRNA to *LacZ* and examined after 7 days. We first examined differentially expressed genes in the four experimental groups and found that *Mrf4* RNAi increased the expression of 677 genes and decreased the expression of 782 genes compared with the control *LacZ* RNAi (fold change >2 and adj. *P*<0.05) (Supplementary Fig. 5 and Supplementary Data 1). The top significant 100 genes comprise 96 upregulated and only 4 downregulated genes (Fig. 2a). As shown in the heatmaps, similar changes were induced by *Mrf4* knockdown in innervated and denervated muscles.

Gene set enrichment analysis (GSEA) revealed that samples transfected with shRNAs to *Mrf4* showed marked enrichment of gene sets involved in muscle contraction, excitation–contraction coupling and energy metabolism (Fig. 2b). A representative sample of muscle genes activated by *Mrf4* knockdown is shown in Supplementary Table 1. Genes coding for the sarcomeric myosin heavy chains (MyHCs) and myosin light chains were among the most upregulated genes, with *Myh1*, *Myh4* and *Myh2*, coding for fast-type MyHC-2X, -2B and −2A, respectively, showing a striking 76-, 45- and 29-fold induction, respectively (Fig. 2c). In addition, genes coding for embryonic (*Myh3*), neonatal (*Myh8*) and extraocular MyHC (*Myh13*) were also significantly upregulated. The upregulation of some of these *Myh* genes was confirmed by qPCR (Fig. 2d and Supplementary Table 2).

A surprising effect of MRF4 knockdown was the upregulation of genes specific for cardiac and smooth muscle tissue, including *Myh6* (α/cardiac MyHC), *Tnni3* (cardiac troponin I), *Myh11* (smooth muscle MyHC), *Myh10* (nonmuscle MyHC IIB, also expressed in smooth muscle), and *Myl9* (smooth muscle myosin light chain 2) (Fig. 2c,d). MyHC-α/cardiac, which is never expressed in extrafusal fibres of leg muscles, was occasionally detected with a specific antibody in some transfected muscle fibres, which also contained MyHC-2A (Fig. 2e). However, cardiac troponin I and MyHC-2B were not detected at the protein level (Supplementary Fig. 6).

### MEF2 target genes are upregulated by *Mrf4* knockdown

**Figure 3:**
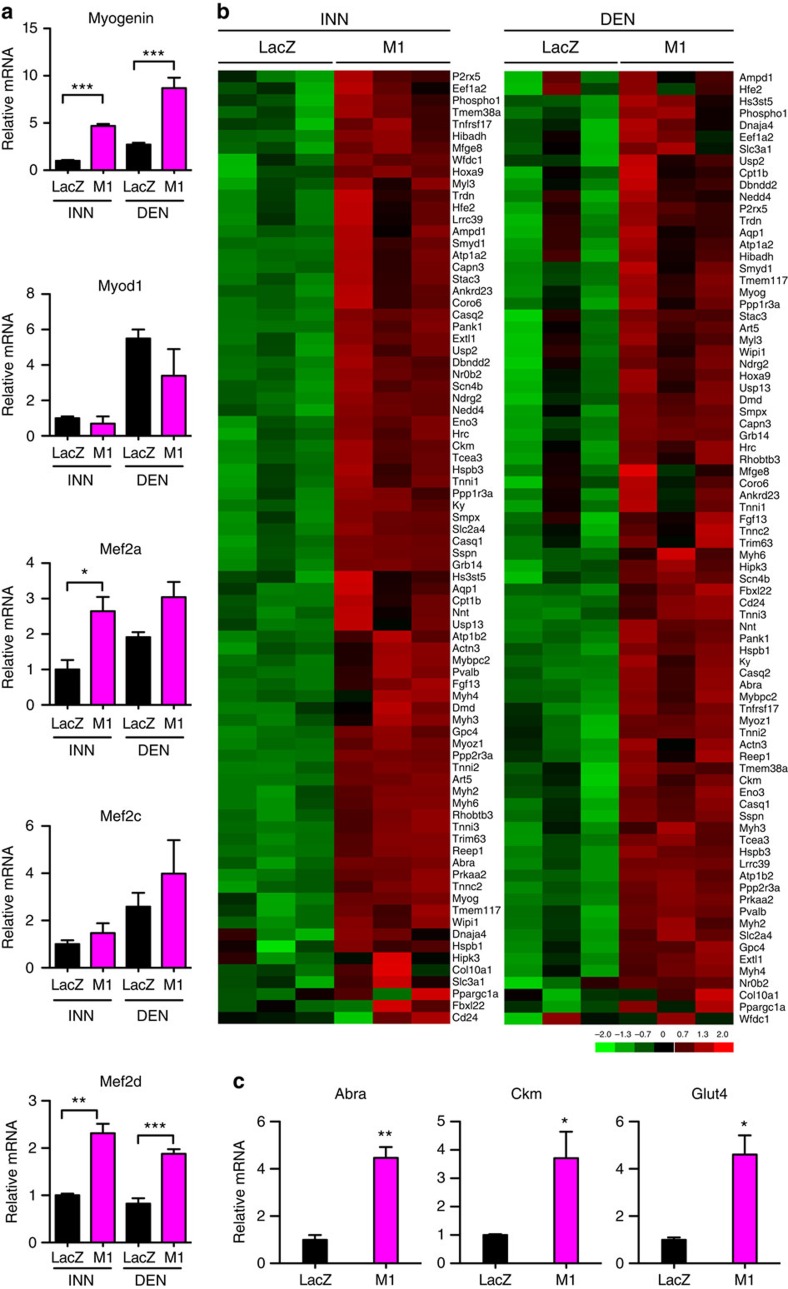
MEF2 targets are upregulated by *Mrf4* knockdown. (**a**) Changes in expression of transcripts coding for myogenin, MyoD, MEF2a, Mef2c and Mef2d induced by MRF4 knockdown, as determined by qPCR (*n*=3, same samples used for microarray analysis in Fig. 2). (**b**) Expression pattern of MEF2 target genes, as determined by crossing the results obtained from GSEA (TRANSFAC) and differential expression analysis. Red and green colours represent relative gene expression levels (red, maximum; green, minimum). (**c**) qPCR shows increased levels of MEF2 targets, including the genes coding for the actin-binding Rho-activating protein (*Abra*), also called STARS, creatine kinase (*Ckm*) and the glucose transporter Glut4, in muscles transfected with *Mrf4* RNAi (M1) compared with control *LacZ* RNAi (*n*=3, same samples used for microarray analysis in Fig. 2). qPCR data are presented as means±s.e.m. Statistical analysis was performed using Student's two-tailed *t*-test (*P<0.05, **P<0.01, ***P<0.001).

GSEA using TRANSFAC showed that gene sets enriched in both innervated and denervated SOL muscles following *Mrf4* knockdown included genes putatively regulated by MYOD and myogenin (Supplementary Table 3). *Myogenin* but not *MyoD* transcripts were significantly upregulated in both innervated and denervated muscles, as shown by qPCR analysis (Fig. 3a). However, the top-ranking gene set upregulated by *Mrf4* knockdown (normalised enrichment score (NES)=2.4; false discovery rate (FDR) <0.0001) was comprised of genes regulated by MEF2 factors (Supplementary Table 3). The transcript levels of *Mef2d* were significantly upregulated by *Mrf4* RNAi in both innervated and denervated muscles and those of *Mef2a* in innervated muscle (Fig. 3a). By comparing the results obtained from GSEA (TRANSFAC) and differential expression analysis we identified a large group of MEF2 targets which were significantly upregulated by *Mrf4* RNAi (Fig. 3b). Increased expression of some of these genes was confirmed by qPCR (Fig. 3c). Upregulated MEF2 targets include a large number of muscle genes encoding myofibrillar and mitochondrial proteins (Supplementary Table 1 and Supplementary Data 1). These results suggest that MEF2 is a major factor in the reprogramming of muscle gene expression induced by *Mrf4* knockdown. Specifically, the fact that *Mrf4* RNAi causes upregulation of MEF2 target genes suggests that MRF4 acts as a repressor antagonizing MEF2 activity. To verify the role of MEF2 factors in mediating the effects of MRF4 knockdown, we examined (i) the existence of a physical interaction between MRF4 and MEF2, (ii) the response of a MEF2 reporter gene to MRF4 knockdown and (iii) the effect of a dominant negative MEF2 mutant on muscle growth induced by MRF4 knockdown.

### MRF4 interacts with MEF2 and represses MEF2 activity

**Figure 4:**
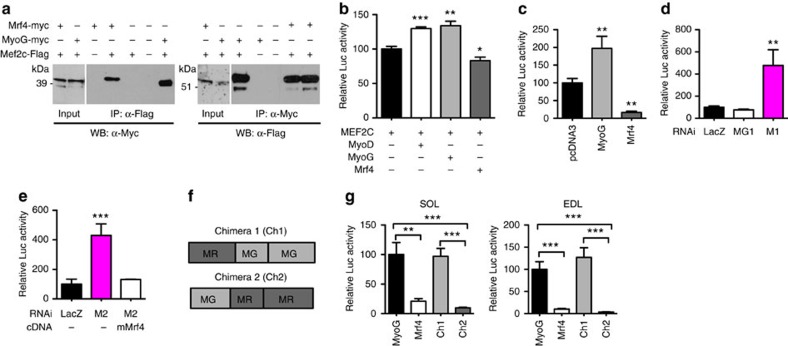
MRF4 physically interacts with MEF2 and inhibits its transcriptional activity. (**a**) HEK-293 cells were transfected with MEF2C-Flag and co-transfected with myc-tagged MRF4 or myc-tagged myogenin (MyoG). Cell lysates were immunoprecipitated with an anti-Flag antibody and resolved by SDS–PAGE, followed by western blotting with anti-Myc antibody. MRF4, like MyoG, is co-immunoprecipitated with MEF2, and MEF2 is co-immunoprecipitated with MRF4 or MyoG. (**b**) HEK-293 cells co-transfected with MEF2C, a MEF2-Luciferase reporter, containing three tandem copies of the MEF2 site linked to luciferase, and either MyoD, myogenin or MRF4 (*n*=3). (**c**) Adult SOL muscle transfected with a MEF2-Luciferase reporter were co-transfected with Mrf4 or myogenin (*n*=4). (**d**) Adult SOL muscle transfected with a MEF2-Luciferase reporter were co-transfected with *Mrf4* RNAi (M1) or *myogenin* RNAi (MG1) (*n*=4). (**e**) Adult SOL muscle transfected with a MEF2-Luciferase reporter were co-transfected with *Mrf4* RNAi (M2) in the presence or absence of the M2-resistant mouse Mrf4 (mMrf4) (*n*=4). (**f**) Structure of two MRF4-myogenin chimeras containing either the N-terminal domain of MRF4 (MR) and the C-terminal domain of myogenin (MG) (chimera 1), or the N-terminal domain of myogenin and the C-terminal domain of MRF4 (chimera 2). (**g**) SOL and EDL muscles were transfected with the MEF2 reporter and co-transfected with either myogenin, Mrf4 or chimeric transgenes (*n*=5). Data are presented as means±s.e.m. from at least three independent experiments. Statistical analysis was performed using Student's two-tailed *t*-test (*P<0.05, **P<0.01, ***P<0.001).

It is known that MyoD and myogenin, when bound to E proteins, can physically interact and synergize with MEF2 factors to induce activation of muscle-specific genes^11^. Importantly, MEF2 and myogenin synergistically activate MEF2 reporters, even in the absence of a functional E-box, while MEF2C can activate E-box-dependent reporters. These findings support the notion that activation of muscle gene expression is mediated by cooperative protein–protein interactions between the two families of transcription factors. However, the interaction between MRF4 and MEF2 has not been explored. To address this issue, we performed co-immunoprecipitation experiments by transfecting HEK-293 cells with plasmids encoding Flag-tagged MEF2C and Myc-tagged MRF4 or Myc-tagged myogenin. As shown in Fig. 4a, co-immunoprecipitation experiments confirm the interaction between MEF2 and myogenin, and support the existence of a similar physical interaction between MEF2 and MRF4.

Next, we studied the effect of MRF4 on a MEF2-dependent reporter. In HEK-293 cells, the reporter was transactivated by myogenin, in agreement with previous studies^11^, but was inhibited by MRF4 (Fig. 4b). In adult skeletal muscle, the MEF2 reporter was likewise transactivated by myogenin but strongly inhibited by MRF4 overexpression (Fig. 4c). An opposite effect was induced by MRF4 knockdown: MEF2 reporter activity was markedly increased by *Mrf4* shRNA (M1), whereas it was unaffected by shRNA against myogenin (Fig. 4d). A similar increase in MEF2 reporter activity was induced by M2 shRNA to rat MRF4 and this effect was completely abrogated by co-transfection with the RNAi-resistant mouse *Mrf4* (Fig. 4e). The increase in MEF2 transcriptional activity induced by MRF4 knockdown was seen in both SOL and EDL muscles and was also obvious in denervated muscles, which showed decreased MEF2 activity relative to control muscles (Supplementary Fig. 7).

To determine whether a specific MRF4 domain is involved in the repression of the MEF2 reporter, we transfected both SOL and EDL muscles with chimeric transgenes containing either the MRF4 N-terminal domain linked to myogenin bHLH and C-terminal domains, or the myogenin N-terminal domain linked to MRF4 bHLH and C-terminal domains (Fig. 4f). Whereas the response to the first chimera is similar to that of myogenin, the second chimera, containing the MRF4 C-terminal domain, has a repressive effect on the MEF2 reporter, similar to that of MRF4 (Fig. 4g). These results suggest that the MRF4-dependent repression of MEF2 transcriptional activity in adult skeletal muscle is associated with the C-terminal domain of MRF4.

### dnMEF2 prevents *Mrf4* RNAi-dependent muscle hypertrophy

**Figure 5:**
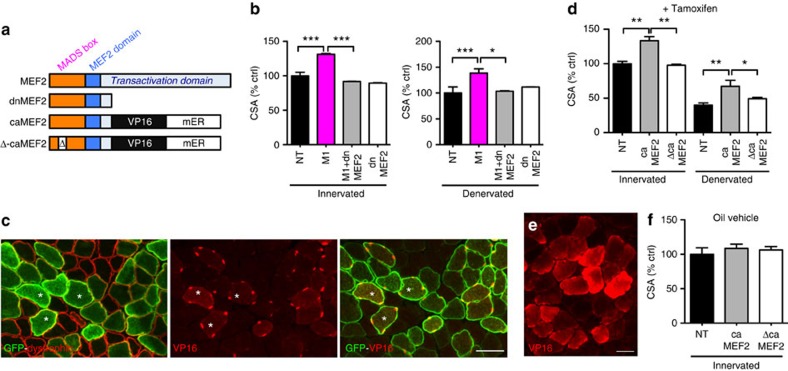
Myofibre hypertrophy is prevented by dnMEF2 and is induced by caMEF2. (**a**) Scheme of the MEF2 constructs used for transfection experiments. (**b**) Innervated or denervated adult SOL muscles were transfected with *Mrf4* shRNA (M1), dominant negative MEF2 (dnMEF2), or co-transfected with both constructs, compared to control muscles (*n*=3). (**c**) Denervated SOL muscle transfected with a constitutively active and inducible MEF2 construct (MEF2-VP16-ER) and co-transfected with GFP. Rats were treated with tamoxifen every other day for 7 days. Transverse sections from denervated transfected SOL stained for dystrophin and co-stained for GFP (left panel) or, on a serial section, for VP16 (central panel) or VP16 and GFP (right panel). Scale bar, 50 μm. (**d**) Innervated and denervated SOL muscles were transfected with caMEF2 or with DNA-binding-deficient Δ-caMEF2. Rats were treated with tamoxifen every other day for 7 days. Cross-sectional areas of transfected fibres normalized to untransfected fibres (*n*=4). (**e**) Innervated SOL muscle were transfected with caMEF2, but rats were treated with oil vehicle rather than tamoxifen. Transverse section was stained for VP16. Scale bar, 50 μm. (**f**) Innervated SOL muscles were transfected with either caMEF2 or Δ-caMEF2, rats were treated with oil vehicle. Cross-sectional areas of transfected fibres normalized to untransfected fibres (*n*=5). Data are presented as means±s.e.m. from at least three independent experiments. Statistical analysis was performed using Student's two-tailed *t*-test (*P<0.05, **P<0.01, ***P<0.001).

To determine whether MEF2 is required for mediating myofibre hypertrophy induced by *Mrf4* RNAi, we used a truncated dominant negative MEF2 (dnMEF2, Fig. 5a), which abrogates MEF2-dependent transcriptional activity in cardiac muscle of transgenic mice^12^ and inhibits muscle-specific gene expression and myotube formation in cultured skeletal muscle cells^13^. The increase in fibre size induced by *Mrf4* RNAi was prevented by co-transfection with dnMEF2 in both innervated and denervated muscles (Fig. 5b). This experiment supports a necessary role of MEF2 in mediating the effect of *Mrf4* RNAi and at the same time supports the existence of a direct link between MEF2 activity and muscle fibre growth in adult skeletal muscle. The following experiment was aimed at validating the existence of this link.

### caMEF2 induces myofibre hypertrophy in adult skeletal muscle

An inducible, constitutively active MEF2 (caMEF2) was used, together with a DNA-binding-deficient version of this construct (Δ-caMEF2), to determine whether an increase in MEF2 transcriptional activity is sufficient to induce myofibre hypertrophy in adult skeletal muscle (Fig. 5a). In agreement with studies in cultured neurons^14^, we found that caMEF2 was localized in myonuclei of transfected myofibres in animals treated with tamoxifen (Fig. 5c). Both innervated and denervated myofibres transfected with caMEF2, but not those transfected with Δ-caMEF2, showed a significant hypertrophy after treatment with tamoxifen (Fig. 5d). In animals treated with oil vehicle rather than tamoxifen, caMEF2 remained sequestered in the cytoplasm and fibre size was unchanged (Fig. 5e,f). These results indicate that MEF2 transcriptional activation is sufficient to induce myofibre hypertrophy in adult skeletal muscle.

### MRF4 knockdown causes HDAC4 nuclear export

**Figure 6:**
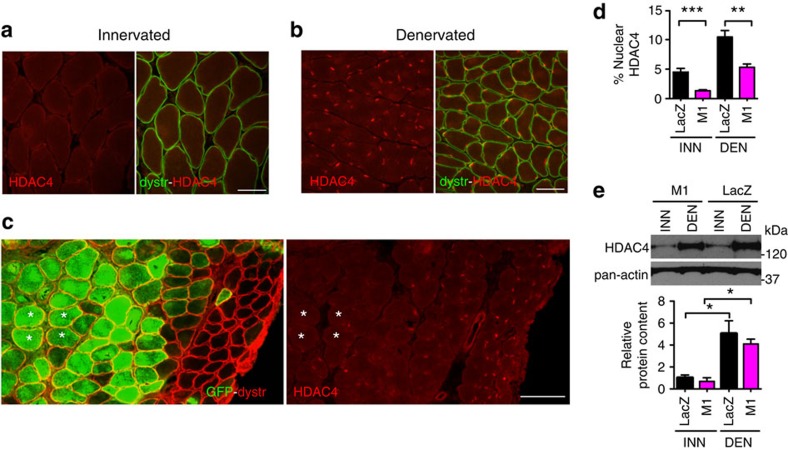
HDAC4 nuclear accumulation is inhibited by *Mrf4* knockdown. (**a**,**b**) HDAC4 distribution in innervated (**a**) and denervated (**b**) SOL muscle. Sections stained for HDAC4 (red) and dystrophin (green). Note nuclear translocation of HDAC4 into myonuclei induced by denervation. Scale bar, 50 μm. (**c**) Denervated SOL muscle transfected with *Mrf4* shRNAs (M1) and co-transfected with GFP. Serial sections were stained for dystrophin and co-stained for GFP (left panel) or for HDAC4 (right panel). Note impaired nuclear accumulation of HDAC4 into transfected hypertrophic myofibres. Four transfected fibres are labelled with asterisks. Scale bar, 50 μm. (**d**) Quantitative analysis of percentage nuclear distribution of HDAC4 in innervated and denervated SOL muscles transfected with shRNAs against *Mrf4* or *LacZ* (*n*=3). (**e**) Western blot of HDAC4 expression in innervated and denervated SOL muscles transfected with *Mrf4* (M1) or *LacZ* shRNAs. Protein content obtained from band quantification normalized to innervated (INN) control muscles is below (*n*=3). HDAC4 is about 5-fold more abundant in denervated compared to innervated muscles, but values are similar in LacZ and M1. Data are presented as means±s.e.m. from at least three independent experiments. Statistical analysis was performed using Student's two-tailed *t*-test (*P<0.05, **P<0.01, ***P<0.001).

To identify the mechanism of MEF2 repression by MRF4, we first asked whether MRF4 acts as a repressor *per se*. However, in agreement with a previous study^15^, no repression was observed in a GAL4 DNA-binding system (Supplementary Fig. 8). Therefore we have considered the possibility that MRF4 may act by affecting the function of a MEF2 repressor. Class IIa histone deacetylases (HDACs), such as HDAC4, are known to bind and repress MEF2 (ref. 16), and their function is controlled by nucleo-cytoplasmic shuttling, with nuclear import leading to MEF2 repression and nuclear export to MEF2 activation^17^. We observed a faint staining of endogenous HDAC4 in both nuclei and cytoplasm of innervated muscle fibres, yet a marked nuclear accumulation of HDAC4 in fibres from denervated muscles (Fig. 6a,b). However, the proportion of nuclear HDAC4 was significantly less in both innervated and denervated myofibres transfected with *Mrf4* RNAi compared to *LacZ* RNAi controls (Fig. 6c,d). The difference was especially striking when comparing transfected and non-transfected regions within the same denervated muscle (Fig. 6c). These data show that HDAC4 nuclear accumulation is markedly decreased by *Mrf4* RNAi. On the other hand, protein levels of HDAC4 remained unchanged, with similar increases observed in both knockdown and control muscles after denervation (Fig. 6e).

### *Mrf4* RNAi and caMEF2 cause mouse muscle hypertrophy

In contrast to the results reported here, no significant effect on muscle fibre size was previously described in an *Mrf4* knockout mouse model^18^ nor in transgenic mice overexpressing a MEF2C-VP16 chimera^19^. To determine whether this discrepancy reflects a species difference or is due to gene inactivation in the adult rather than in the embryo, we performed electroporation experiments in adult mouse muscles. As shown in Supplementary Fig. 9a,b, the two major effects of MRF4 knockdown, namely myofibre growth and activation of the MEF2 reporter, were also evident in transfected adult mouse muscles. Myofibre hypertrophy was also induced by caMEF2 (Supplementary Fig. 9c,d). These results indicate that muscle fibre size is also controlled by the MRF4–MEF2 axis in adult mouse muscles.

We also performed chromatin immunoprecipitation (ChIP) assays on mouse muscles to determine whether endogenous MRF4 is present on MEF2-binding sites in muscle gene promoters. ChIP assays were performed in denervated muscles, a condition where a greater MRF4 binding would be predicted. As shown in Supplementary Fig. 10, MRF4 was readily detected on the promoters of MEF2 target genes, *Abra* and *Glut4*. Importantly, the promoter of *Abra* does not contain any E-box in the amplified region. The specificity of this result is supported by the finding that no MRF4 binding was detected in muscles from *Mrf4* knockout mice.

## Discussion

The myogenic regulatory factors are well known to play key roles during embryonic and fetal myogenesis, and in satellite cell-mediated regeneration in adult muscle, however, their role in adult muscle fibres is unknown. This is an especially important issue in the case of MRF4, the expression of which undergoes a marked upregulation during late fetal and postnatal development. The unexpected findings reported here provide a novel interpretation of the role of MRF4 in adult muscle fibres. We show firstly that *Mrf4* RNAi causes fibre hypertrophy and prevents denervation-induced atrophy, indicating that MRF4 acts as a negative regulator of muscle growth, and secondly that this effect is mediated by the transcriptional activation function of MEF2 which acts as a positive regulator of growth. Our data lead to a model in which in normal homeostasis the genes required for the hypertrophic response are held in check by MRF4 working to repress activation by MEF2. When this balance is disturbed and the repression by MRF4 is lifted, MEF2 drives the transcription of the gene set required for hypertrophy, leading to increased protein synthesis and fibre growth. Myofibre hypertrophy induced by *Mrf4* RNAi occurred in transfected fibres from both fast and slow muscles. A similar effect was observed in regenerating muscle, in which muscle fibre growth was accelerated, and in denervated muscles, in which muscle atrophy was prevented. These results are somewhat surprising as no obvious effect on fibre size in fast and slow limb muscles was described in the one *Mrf4* knockout allele compatible with survival^18^. This discrepancy may be due to compensatory effects that arise when the gene is deleted from early embryonic stages and thus an entire transcriptional programme is altered throughout development. Indeed, it was previously reported that muscle fibre growth during regeneration is retarded in a line of transgenic mice overexpressing MRF4 (ref. 20). The increase in muscle growth induced by *Mrf4* RNAi is due to elevated protein synthesis, as determined by the increased amount of puromycin-labelled peptides, in agreement with other models of muscle hypertrophy^9^. However, further studies, including analyses of protein degradation pathways, are required to define the changes in protein turnover induced by *Mrf4* RNAi and the signalling pathways involved. Indeed, we found that the ubiquitin ligase MURF1, which is usually associated with muscle atrophy, is also upregulated by *Mrf4* RNAi at the transcript level (not shown). This is not surprising, as other models of muscle hypertrophy are accompanied by an increase in both protein synthesis and degradation^21^, possibly in relation with the reprogramming of gene expression and remodelling of muscle structure induced by muscle growth.

MEF2 transcription factors are known to be essential for myogenesis both *in vitro*^13^,^22^ and *in vivo* during development^23^ and during muscle regeneration^22^. However, MEF2 factors have not been associated with muscle hypertrophy in adult skeletal muscle, although they are known to be involved in cardiac muscle hypertrophy^24^. Several lines of evidence support a major role for MEF2 factors in mediating the effect of MRF4 knockdown on muscle hypertrophy. First, the top-ranking gene set enriched in both innervated and denervated muscles transfected with MRF4 shRNA is comprised of genes regulated by MEF2 factors. Second, co-immunoprecipitation assays support the existence of a physical interaction between MRF4 and MEF2 factors. Third, MEF2 transcriptional activity is strongly stimulated by *Mrf4* RNAi and repressed by MRF4 overexpression, whereas opposite effects are produced by myogenin overexpression, in agreement with previous studies in cultured muscle cells^11^. Finally, the increase in myofibre size induced by *Mrf4* RNAi is prevented by dominant negative MEF2, while constitutively active MEF2 is able to induce myofibre hypertrophy in adult muscle. Thus MEF2 appears to be both necessary and sufficient to mediate *Mrf4* knockdown-induced muscle hypertrophy. Taken together, our results suggest that MRF4, unlike MyoD and myogenin, acts to repress MEF2 transcriptional activity rather than to stimulate it. Previous studies showed that MRF4, unlike MyoD and myogenin, is not able to transactivate the promoters of many muscle genes, in cultured mammalian muscle cells^25^,^26^.

The results of the present study show that MRF4 RNAi in adult muscles leads to an increase in MEF2 transcriptional activity with upregulation of both fast and slow skeletal muscle genes and, surprisingly, also cardiac and smooth muscle genes. MEF2 is known to be essential for skeletal, cardiac and smooth muscle myogenesis^27^. In developing zebrafish skeletal muscles, MEF2 factors were shown to control the expression of both fast and slow myosins and other thick filament proteins^28^. In mice, muscle-specific knockout of *Mef2c* or *Mef2d* causes a partial slow-to-fast switch in mouse soleus, whereas transgenic mice overexpressing a hyperactive *Mef2c* show an increased proportion of slow fibres^19^. On the other hand, both fast and slow muscle genes were downregulated in cultured satellite cells after triple knockout of *Mef2a*, *c* and *d* genes using tamoxifen-inducible Cre recombinase driven by the satellite cell-specific Pax7 promoter^22^. Indeed, the most downregulated *Myh* genes in MEF2-deficient muscle cells were *Myh4* and *Myh1*, coding for the fast MyHC-2B and 2X, respectively, which are also among the most upregulated genes after MRF4 knockdown. The finding that cardiac and smooth muscle genes are also induced by MRF4 knockdown could reflect the fact that the potential role of MEF2 as a global regulator of multiple muscle differentiation programs is restrained by MRF4 in adult skeletal muscle.

We have shown that the muscle-specific transcription factor MRF4 inhibits muscle growth in both regenerating and adult skeletal muscle, and have provided evidence that this effect is mediated by a repressive action of MRF4 on the positive transcriptional activity of MEF2. MRF4 is not a repressor *per se*, therefore we suggest that it acts by affecting the function of a MEF2 repressor. HDAC4 is the best characterized MEF2 repressor, which shuttles between the cytoplasm and the nucleus and once in the nucleus associates with MEF2 factors and represses their transcriptional activity, whereas HDAC4 nuclear export leads to MEF2 activation^16^,^17^,^29^. HDAC4 appears to be involved in MRF4-dependent MEF2 repression because its nuclear localization in adult skeletal muscle is impaired by *Mrf4* knockdown. It is known that HDAC4 enzymatic activity is dependent on a multiprotein complex containing class I HDACs, like HDAC3, and co-repressors, such as NCoR1 (nuclear receptor co-repressor 1)^30^. Interestingly, a muscle-specific knockout of *NCoR1* leads to the activation of MEF2 and the induction of muscle hypertrophy, among other phenotypes^31^. The demonstration that MRF4 binds MEF2 and is found on MEF2 sites in muscle gene promoters suggests that MRF4 is a key component, together with HDAC4 and NCoR1, of the multiprotein repressive complex that regulates the activity of the pro-hypertrophic transcription factor MEF2. The loss of MRF4 might lead to a destabilization of the complex with a consequent increase in MEF2 transcriptional activity, which may well be mediated by increased acetylation of MEF2 consequent upon the relocation of HDAC4 to the cytoplasm. The interactions between MRF4 and the other components of the repressive complex and the mechanism leading to HDAC4 nuclear export remain to be established.

The suggested role of HDAC4 in mediating MRF4-dependent derepression of MEF2 activity differs from the previously described role of HDAC4 in controlling neurogenic muscle atrophy via myogenin. Denervation causes upregulation and nuclear accumulation of HDAC4 (ref. 32) and HDAC4 knockdown prevents denervation-induced myogenin upregulation, acting via the corepressor Dach2 (refs 33, 34). The finding that mice lacking both HDAC4 and HDAC5 in skeletal muscle are resistant to muscle atrophy upon denervation supports the notion that the HDAC4–Dach2–myogenin pathway controls denervation atrophy^8^. However, it is unlikely that this pathway is implicated in the effect of *Mrf4* knockdown because the expression of myogenin is increased but that of HDAC4 is unchanged by *Mrf4* knockdown in both innervated and denervated muscle. In addition, the changes in gene expression induced by *Mrf4* knockdown are very similar in innervated and denervated muscle. Finally, another study suggested an alternative pathway, dependent on HDAC4-MAP kinase crosstalk and independent of myogenin induction, to connect HDAC4 to the muscle atrophy programme^35^. This pathway is also unrelated to the MRF4–MEF2 axis described here, because it involves the deacetylation and activation of a key MAP3 kinase, MEKK2, whereas the conserved histone deacetylase domain of HDAC4 is completely dispensable for MEF2 repression^36^.

Our finding that muscle growth can be induced in adult skeletal muscle by lifting MRF4-mediated repression and thus activating MEF2 transcriptional activity might provide a new strategy to combat muscle wasting. We have shown here that denervation atrophy can be inhibited by activated MEF2 and it will be of interest to determine whether this also occurs in other conditions of muscle atrophy, including sarcopenia and cachexia. Current treatments for muscle wasting, such as those based on the inhibition of the type-II activin receptors, are non-ideal, given the lack of tissue specificity and the contrasting effects of the ligands binding these receptors. Indeed, recent studies indicate that the disruption of the activin pathway may be potentially deleterious in cardiac and skeletal muscle^37^,^38^. By contrast, MRF4 is exclusively expressed in skeletal muscle, not in heart or any other tissue, thus providing a unique tissue-specific target. Pharmacological treatments aimed at downregulating MRF4 expression, disrupting MRF4–MEF2 interactions or modulating corepressor function may thus be viable approaches to maintain muscle mass and prevent muscle wasting.

## Methods

### Animals and *in vivo* experiments

Experiments were performed in 6-week-old male Wistar rats (about 150 g) or 2-month-old male CD1 mice (about 30 g). EDL and soleus muscles were exposed in anaesthetised animals and injected with plasmid DNA (30 μg in 50 μl saline). Injection was followed by electroporation with stainless steel electrodes connected to an ECM830 BTX porator (Genetronics) with the following settings: six pulses of 20 ms each and 200-ms interval, voltage adjusted to the thickness of the leg (220 V cm^−1^). For transfection of regenerating muscles, plasmid DNA (30 μg in 20% sucrose) was directly injected into the muscles at day 3 after bupivacaine treatment without electroporation^39^. Denervation was achieved by cutting the sciatic nerve high in the thigh. Muscles were removed 7 or 14 days after transfection, frozen in isopentane cooled in liquid nitrogen, and stored at −80 °C. Activation of transfected MEF2-VP16-ER was induced by injecting tamoxifen 30 mg kg^−1^ in sunflower oil every other day for seven days. For puromycin experiments and microarray analyses, soleus muscles were chosen because they consistently showed a much greater efficiency of gene transfer after electroporation compared to EDL muscles. We first screened transfected soleus muscles by GFP fluorescence to identify those muscles with the highest transfection efficiency and discarded the proximal and distal portions close to the tendons, which are generally poorly transfected. The top three transfected muscles from each of the four groups, that is, innervated and denervated M1, and innervated and denervated *LacZ*, were thus selected. All experimental procedures were performed according to the European Commission guidelines and were approved by the local ethical committee and the relevant Italian authority (Ministero della Salute, Ufficio VI), in compliance of Italian Animal Welfare Law (Law n 116/1992 and subsequent modifications) and complying with the Directive 2010/63/EU of the European Parliament. Animals were anaesthetized by ip injection of a mixture of Zoletil 100 (Zolazapam and Tiletamine, 1:1, 10 mg kg^−1^) and Xilor (Xilazine 2%, 0.06 ml kg^−1^), or by using an isoflurane vaporizer maintained at 2% isoflurane, 1 l m^−1^ oxygen.

### Cell culture and transfection

HEK-293 cells (ATCC) were cultured in DMEM with 10% fetal bovine serum in a humidified incubator at 37 °C with 5% CO_2_ and transfected using Lipofectamine2000 (Invitrogen) and following the procedure recommended by the manufacturer. Cultures were tested for mycoplasma contamination. Cell lysates were prepared and analysed 48 h after transfection.

### Muscle and cell lysate preparation

For total muscle lysates, about 25 sections (20 μm thick) of muscles were lysed in Laemmli buffer (10% w/v glycerol, 5% w/v β-mercaptoethanol, 2.3% w/v SDS, 62.5 mM Tris-HCl containing ‘complete' protease inhibitor cocktail (Roche, one tablet per 1 ml)). Homogenates were sonicated for 5 s. HEK-293 cells were lysed in RIPA buffer containing ‘complete' protease inhibitor cocktail.

### Western blotting and co-immunoprecipitation assays

Lysates (30 μg) were heated in SDS sample buffer and subjected to SDS–PAGE and electrotransfer to nitrocellulose membranes. Membranes were pre-incubated for 16 h at 4 °C in 50 mM Tris, pH 7.5, 150 mM NaCl, and 0.1% Tween (TBST) containing 5% skimmed milk. Antibodies were diluted in TBST. Detection of proteins was performed using horseradish peroxidase-conjugated secondary antibodies (Bio-Rad) and the enhanced chemiluminescence reagent (Pierce Biotechnology, USA). For co-immunoprecipitation experiments, Myc-tagged MRF4 or myogenin were transfected in HEK-293 cells in presence or absence of Flag-tagged MEF2C. All constructs were co-transfected with a plasmid coding for E47 protein and cells were lysed after 24 h. Immunoprecipitations with anti-Myc or anti-Flag antibodies were performed on cell lysates with Immunocruz IP/WB Optima System (Santa Cruz) using the procedure recommended by the manufacturer.

Uncropped scans of all the autoradiographies shown in this work are reported in Supplementary Fig. 11.

### *In vivo* protein synthesis assays

Analysis of protein synthesis was performed using a nonradioactive technique as described^40^. In brief, rats were anaesthetized and injected i.p. with 0.04 μmol g^−1^ puromycin exactly 30 min before removal of muscles. Transfected muscles were first examined by GFP fluorescence to identify regions showing the best gene transfer. Muscle homogenates were processed for immunoblotting analysis using the 12D10 monoclonal antibody, specific for puromycin^10^.

### Immunofluorescence and fibre size measurement

About 10 μm thick transversal muscle sections were processed for immunofluorescence using standard conditions. Immunofluorescence was performed on unfixed muscle cryosections for MyHC and cardiac troponin I staining. For MRF4 staining a fixation step with 4% paraformaldehyde (PFA), 10 min at room temperature, was performed before primary antibody incubation. Sections were permeabilized with 0.2% Triton X-100 for 10 min at room temperature. DyLight488-labelled secondary antibodies (Jackson laboratories) were used. Images were collected with an epifluorescence Leica DM5000B microscope equipped with a DFC 300FX camera. Cross-sectional area of individual fibres was manually outlined using ImageJ NIH (http://imagej.nih.gov/ij). For HDAC4 localization, sections of muscles transfected with M1 and GFP were fixed in PFA and stained for HDAC4 and dystrophin. Transfected fibres and their myonuclei were identified by GFP and DAPI staining. The HDAC4 fluorescence intensity of pixels for each transfected fibre cross-section were quantitated using ImageJ NIH software. For each fibre the percentage of nuclear HDAC4 was calculated by the ratio of the fluorescence intensity of endogenous HDAC4 in myonuclei divided by the total fluorescence intensity of the same fibre (nuclei/cytoplasm+nuclei). Results are expressed as the mean±s.e.m.

### Antibodies

The following antibodies were purchased from Santa Cruz BioTechnology: Myf6 C-19 rabbit polyclonal (sc-301 X; 1:500); myogenin monoclonal (sc-12732; 1:1,000); β-Tubulin rabbit polyclonal (sc-9104; 1:5,000), green fluorescent protein rabbit polyclonal (sc-8334; 1:500), HDAC4 rabbit polyclonal (sc-11418; 1:500), VP16 monoclonal (sc-7545; 1:100). The c-myc 9E10 monoclonal antibody (prod. Num 116672030001; 1:500) was from Roche, the flag M2 (F1804; 1:2,000) monoclonal antibody and the anti-puromycin 12D10 (MABE343; 1:5,000) monoclonal antibody were from Merk-Millipore, and anti-dystrophin (MANDRA 1) from Developmental Studies Hybridoma Bank (DSHB; 1:1,000). Green Fluorescent Protein rabbit polyclonal (for immunofluorescence staining) was purchased from Invitrogen (A11122; 1:200). The following monoclonal antibodies are homemade and are distributed by DSHB: SC-71 (MyHC-2A; 1:100), BF-F3 (MyHC-2B; 1:100), BF-G6 (MyHC-emb; 1:200), BF-B6 (MyHC-neo; 1:500), BA-G5 (MyHC-α cardiac; 1:500), TI-1 (troponin I cardiac 1:500)^41^,^42^,^43^,^44^,^45^.

### RNAi-mediated gene silencing

The sequences of *Mrf4* and *Myog* genes were retrieved and analysed. The target sequences were selected on unique regions with respect to the others MRFs. Target oligos were designed by using the criteria defined by Reynolds *et al*.^46^. We selected at least 4 oligos per gene on the basis of a specificity screening performed with BLAST analysis. The selected oligos were cloned into the pSUPER vector. We co-transfected the epitope-tagged cDNA together with each pSUPER construct. As a control, we used pSUPER constructs targeting LacZ. To exclude that the observed downregulation of Mrf4 or MyoG could be due to off-target effects, we selected at least two sequences with high silencing efficiency for each gene, and rescue experiments were performed by co-transfecting RNAi-resistant cDNAs. The sequences used are indicated below with the corresponding position on the targeted *Mrf4* or *MyoG* sequence:

Mrf4 M1: GCAAGACCTGCAAGAGAAA (National Center for Biotechnology Information (NCBI) Reference Sequence NM_ 008657).

Mrf4 M2: GCGAAAGGAGGAGGCTTAA (National Center for Biotechnology Information (NCBI) Reference Sequence NM_ 013172).

MyoG MG1: CCATGCCCAACTGAGATTG (National Center for Biotechnology Information (NCBI) Reference Sequence NM_ 017115).

MyoG MG2 GAGAAGCACCCTGCTCAAC (National Center for Biotechnology Information (NCBI) Reference Sequence NM_ 017115).

### RNA isolation and qPCR

For quantitative real-time-PCR assays, total RNA was purified using SV Total RNA Isolation (Promega, Madison, WI, USA) and its integrity was assessed by capillary electrophoresis (Agilent, Santa Clara, CA, USA). RNA (400 ng) was converted to cDNA using random primers and Superscript III (Invitrogen, Carlsbad, CA, USA). Amplification was carried out in triplicates with a 7900HT Fast Real Time PCR System (Applied Biosystems) using the Fast SYBR Green RT-PCR kit (Applied Biosystems) and a standard 2-step protocol. The primers specific for each gene were designed and analysed with Primer3 (freeware) and Vector NTI (Invitrogen, freeware). Identity of the amplicons was confirmed by their dissociation profiles and gel analysis. Quantitative PCR standard curves were constructed by using serial dilutions of muscle cDNAs, using at least four dilution points and the efficiency of all primer sets was between 1.8 and 2.2. The data were normalized against *Rplp0* housekeeping gene. Primers are listed in Supplementary Table 2.

### Plasmids

The plasmids were prepared using Qiagen Maxiprep Kit (Invitrogen) according to the manufacturer's instructions. pSUPER vector in which shRNAs were inserted under the control of the polymerase-III H1-RNA gene promoter was from Oligoengine. pcDNA3 was from Invitrogen, Tk-Renilla from Promega. Rat Mrf4 and MyoG were from Addgene; human Mrf4 from Origene. Myc-Mrf4 was obtained by subcloning mouse Mrf4 in pcDNA3.1Myc-HisB. Mef2-Flag and dominant negative MEF2 (dnMEF2) were kindly provided by Windt. The Mef2 reporter plasmid, kindly provided by Rhonda Bassel-Duby, contains three copies of the Mef2 element from the muscle desmin gene promoter, followed by the luciferase gene. Mrf4-MyoG chimeras were a kind gift of Moss^47^. MEF2-VP16-ER (caMEF2) and MEF2ΔDBDVP16-ER (Δ-caMEF2: caMEF2 with deleted DNA-binding domain) plasmids were kindly provided by M. Greenberg^14^.

### Luciferase assay

For luciferase assay we used the Dual luciferase kit E1960, Promega Corp., Madison WI, USA. Muscles were crushed with a pestle and mortar cooled with liquid nitrogen. Powder was weighted and added with 2.5 μl of lysis buffer for each mg of tissue. Lysates were frozen with liquid nitrogen and thawed at 4 °C twice. Lysates were then centrifuged for 20 min at 13,000 r.p.m. at 4 °C, and supernatants were collected. Ten microliter of supernatant were analysed according to the manifacturer's instructions.

### Chromatin immunoprecipitation and real-time qPCR

ChIP-qPCR of mouse gastrocnemius muscle from wild-type and *Mrf4* knockout^18^ mice was performed with sonicated nuclear extract prepared from formaldehyde-crosslinked tissue according to Nelson *et al*.^48^ using anti-MRF4 antibody (Proteintech 11754) or rabbit IgG (Santa Cruz 2027x). Immunoprecipitated DNA was decrosslinked, purified and used for quantitative real-time PCR. Primer sequences used are the following: Abra_Promoter_FW CCAGCTAAAGGGGAATGTGGT; Abra_Promoter_RV TAGTTTCCACCGTCACAGGC; Glut4_Promoter FW GACACCCGGGACCTGACATT; Glut4_Promoter RV CATGTACTTGCCAGGGTACGG; Hprt_intron_FW CAACCACTTACTTAGAGGTACT; Hprt_intron_RV TTAGCAATATGGACTGTGAGGG.

### Microarray analysis and computational tools

For microarray experiments, three biological replicates for each group, including innervated and denervated MRF4 RNAi (M1) and innervated and denervated LacZ RNAi, were hybridized on Affymetrix Rat Genome 230 2.0 arrays. Isolated total RNA samples were processed as recommended by Affymetrix (Affymetrix, Inc., Santa Clara, CA). All data analyses were performed in R (version 2.15.0) using Bioconductor libraries (BioC 2.7) and R statistical packages. Probe level signals were converted to expression values using robust multi-array average procedure RMA^49^ of Bioconductor *affy* package and Brainarray rat2032rnentrezg custom CDF files (http://brainarray.mbni.med.umich.edu/Brainarray/Database/CustomCDF). Statistically significant differences in gene expression between M1 and LacZ samples were determined using the empirical Bayes moderated *t*-test in the Bioconductor *limma* package^50^. In *limma*, we selected differentially expressed genes setting a fold change≤±2 and an adj. *P* value≤0.05 after multiple testing corrections^51^. The expression of a select panel of genes was validated using real time quantitative reverse transcription PCR (qRT-PCR). We used Gene Set Enrichment Analysis (GSEA, http://http://www.broadinstitute.org/gsea^52^) to determine whether *a priori* defined sets of genes showed statistically significant differences between the M1 and LacZ samples. Enrichment analysis was performed on gene sets derived from the Biological Process Ontology (C5, CC: GO cellular component) and from the TRANSFAC transcription factor binding site (C3, TFT: transcription factor targets) categories of the Molecular Signatures Database (http://www.broadinstitute.org/gsea/msigdb/index.jsp). The GO biological set was supplemented with a list of sarcomeric myosins specifically expressed in skeletal muscle (Supplementary Table 4). Gene sets were defined as significantly enriched if the FDR was<5% when using Signal2Noise as metric and 1,000 permutations of gene set.

### Statistical analysis

Data are presented as mean±s.e.m. Statistical significance was determined using two-tailed Student's *t*-test with a significance threshold of 0.05 (*P<0.05; **P<0.01; ***P<0.001). Tests were applied upon verification of the test assumptions (for example, normality). For the measurement variables used to compare different conditions, the variance was similar between the groups. Animals were randomly allocated to the different experimental groups. Investigators were blinded when transfecting animals and when assessing the outcome of experiments. Exclusion criteria for rats/mice were pre-determined: animals were excluded from analysis in case of cannibalism, sickness, or poor transfection efficiency (less than 10 transfected fibres). Minimum sample size was determined using size power analysis methods for *a priori* determination, based on the standard deviation and effect size previously obtained using the same experimental methods employed in the study. We used G*Power Software with alpha=0.05 and a power of 0.8 to determine the needed sample size.

### Data availability

Microarray data are deposited in the NCBI Gene Expression Omnibus (GEO) under accession code GSE67069. The data that support the findings of this study are available from the corresponding author upon request.

## Additional information

**How to cite this article:** Moretti, I. *et al*. MRF4 negatively regulates adult skeletal muscle growth by repressing MEF2 activity. *Nat. Commun.* 7:12397 doi: 10.1038/ncomms12397 (2016).

## Supplementary Material

Supplementary Information Supplementary Figures 1-11 and Supplementary Tables 1-4.

Supplementary Data 1 Full list of differentially expressed genes (up- and downregulated compared to control), derived from microarray analysis of innervated and denervated SOL muscles transfected with shRNA to Mrf4 (M1 sequence).
