# Supplementary material for: MRF4 negatively regulates adult skeletal muscle growth by repressing MEF2 activity
Source: Nat Commun. 2016 Aug 3;7:12397. doi: 10.1038/ncomms12397 (PMC4976255; doi:10.1038/ncomms12397)
Supplement: Supplementary Information — Supplementary Figures 1-11 and Supplementary Tables 1-4. [file ncomms12397-s1.pdf]

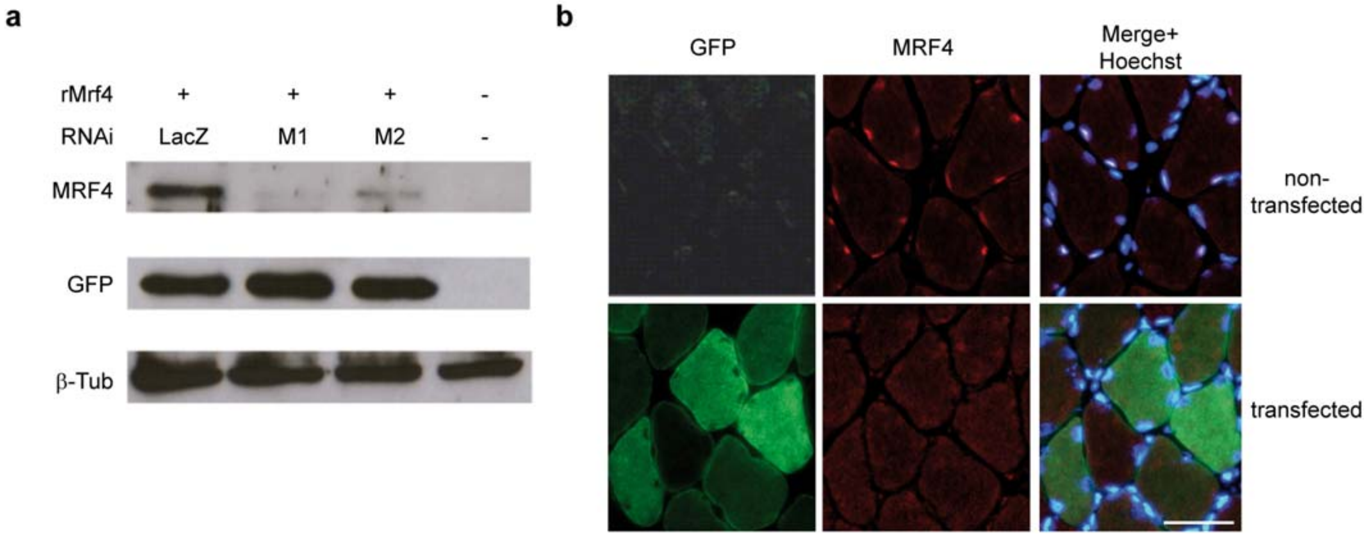

Supplementary Figure 1. **Validation of MRF4-specific shRNA constructs**. (**a**) HEK 293 cells were transfected with rat MRF4 and co-transfected with a control pSUPER vector that generates shRNAs against LacZ, or two vectors that generate shRNAs against MRF4 (M1 and M2). GFP was co-transfected to determine transfection efficiency. Western blotting with anti-MRF4 and anti-GFP show efficient MRF4 knockdown induced by M1 and M2. (**b**) Rat soleus transfected with M1 and co-transfected with GFP. Whereas untransfected GFP negative fibers show nuclear staining with anti-MRF4 antibody (upper panels), transfected GFP positive muscle fibers are unreactive for MRF4 (lower panels). Scale bar 50 μm.

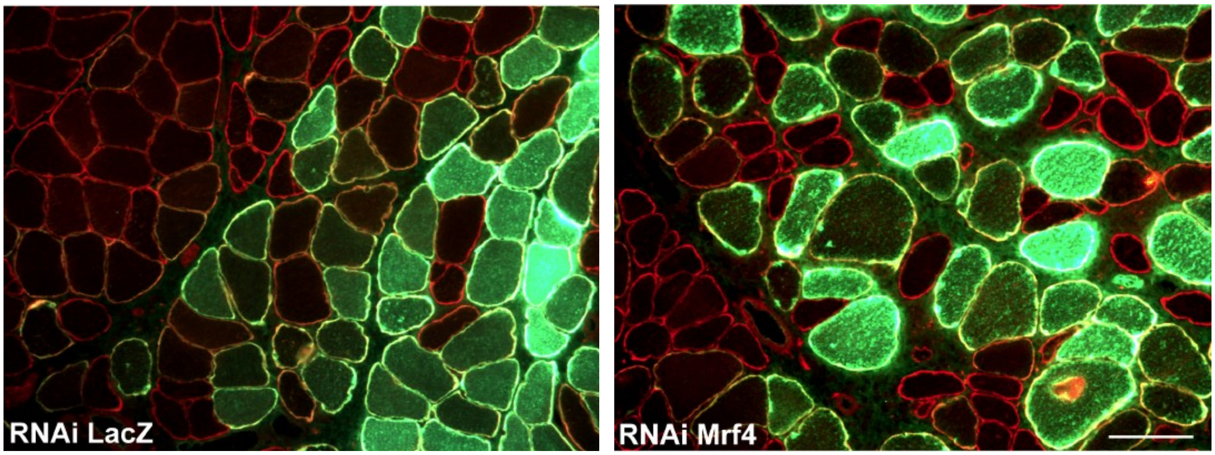

Supplementary Figure 2. Fiber size changes in transfected versus non-transfected fibers in the same muscle. Transverse sections of soleus transfected with either LacZ shRNAs or Mrf4 shRNAs (M1) and examined at 14 days after transfection. Transfected fibers identified by GFP fluorescence, section stained for dystrophin (red) to visualize fiber profiles. Note larger fiber size in muscle fibers transfected with Mrf4 shRNAs compared to neighboring untransfected fibers. Scale bar 100 µm.

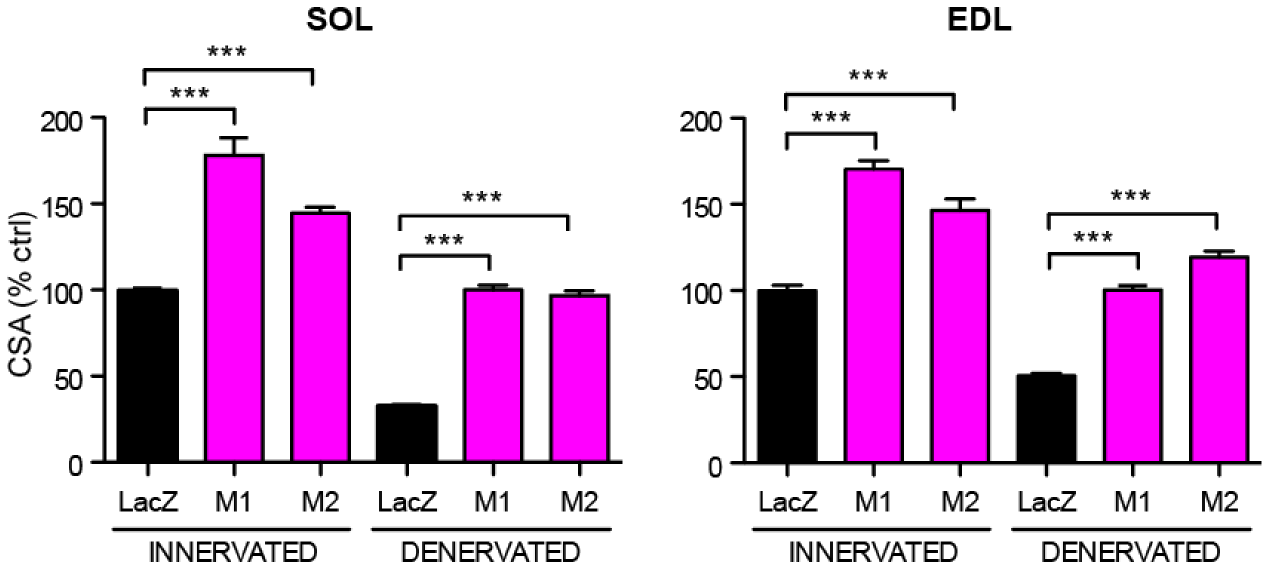

Supplementary Figure 3. Effect of *Mrf4* RNAi in adult rat muscle at 14d after transfection. Cross-sectional areas of muscle fibers in innervated and denervated SOL and EDL muscles transfected with M1 or M2 and co-transfected with GFP. Values normalized to fibers transfected with LacZ shRNAs in innervated SOL or EDL. At least 4 muscles per group were analyzed, data are presented as means±SEM from at least three independent experiments. Statistical analysis was performed using Student's two-tailed *t*-test (*p<0.05, **p<0.01, ***p<0.001).

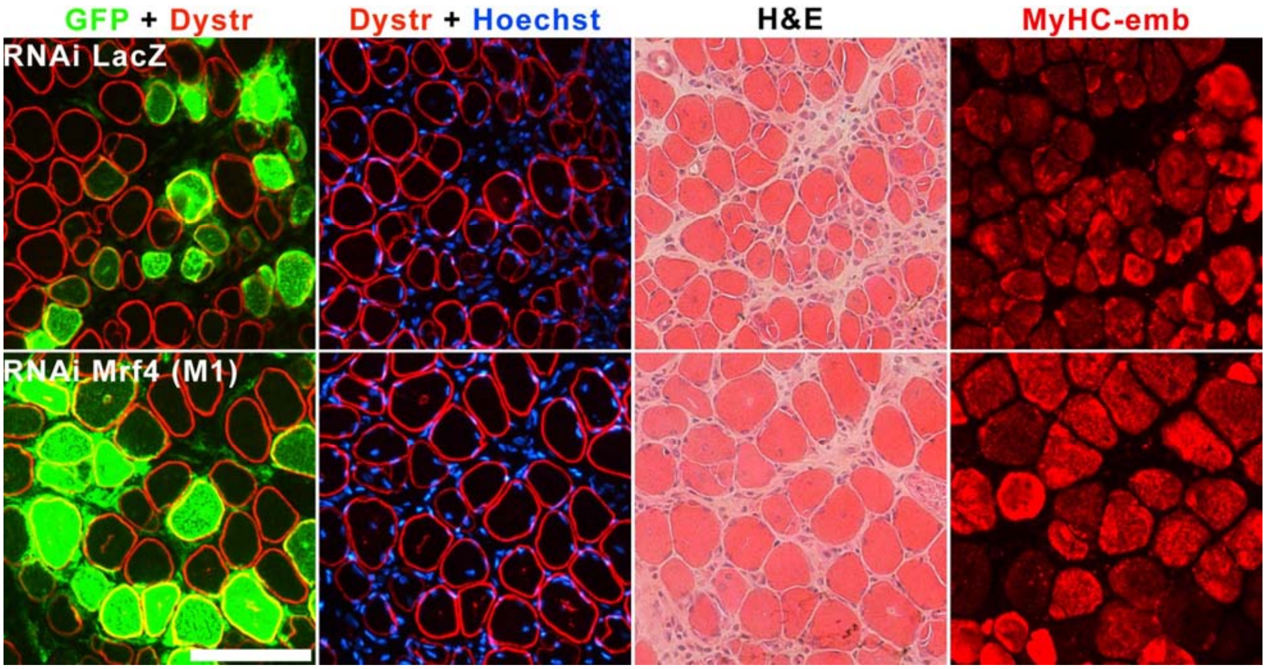

Supplementary Figure 4. Effect of Mrf4 RNAi in regenerating muscle. Rat SOL was co-transfected at day 3 after bupivacaine-induced muscle injury with GFP and Mrf4 or LacZ shRNAs and examined 7 days later. Serial sections were stained with H&E, and with anti-GFP (to visualize transfected fibers), anti-dystrophin (to visualize fiber profiles), Hoechst (to visualize nuclei), and anti-MyHC-emb. Scale bar 100 µm.

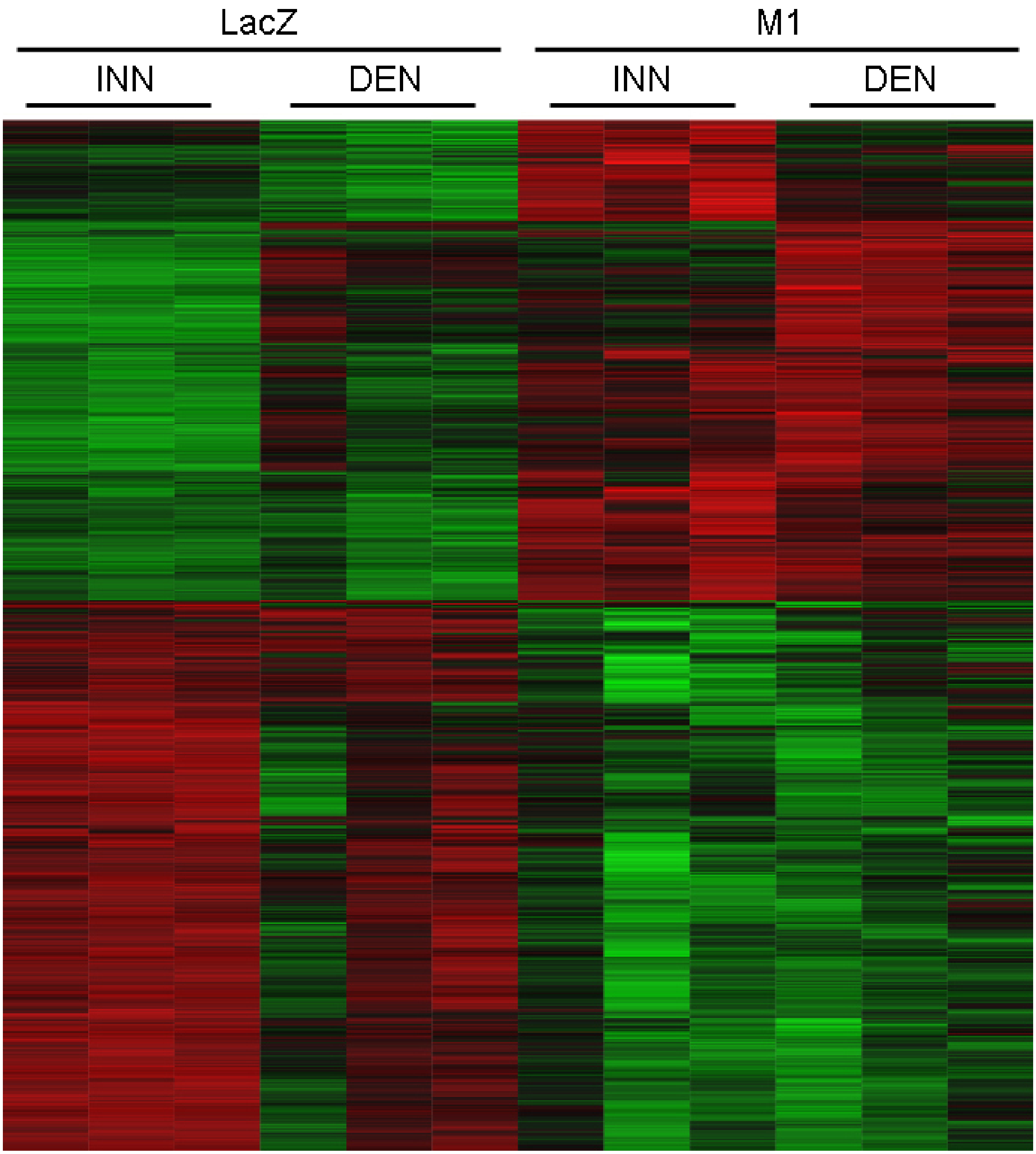

Supplementary Figure 5. Effect of *Mrf4* RNAi on global gene expression profile. Heatmap representing hierarchical clustering of differentially expressed genes (p-value < 0.05; fold change > 2) between *Mrf4* RNAi (M1) and *LacZ* RNAi innervated and denervated muscles. Each lane corresponds to one animal, 3 animals per group were analyzed.

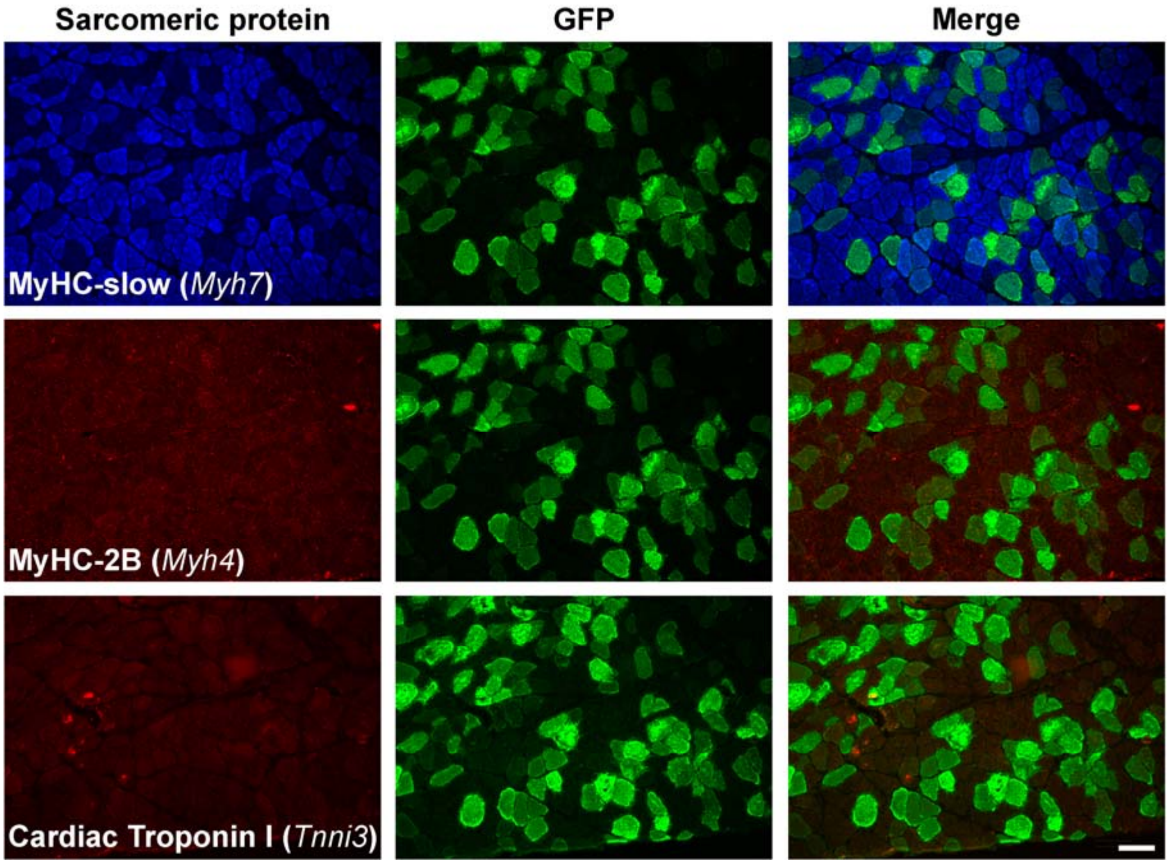

Supplementary Figure 6. Changes in protein levels of selected sarcomeric proteins induced by *Mrf4* RNAi. SOL muscle transfected with *Mrf4* shRNAs and examined at 7 days after transfection by immunofluorescence with anti-MyHC slow, antiMyHC 2B anti-cardiac troponin antibodies. Note that MyHC-2B and troponin I, whose transcripts are upregulated by *Mrf4* knockdown (see Fig. 2c and Supplementary Table 1), are not detectable at the protein level in transfected muscle fibers. Scale bar 100 µm.

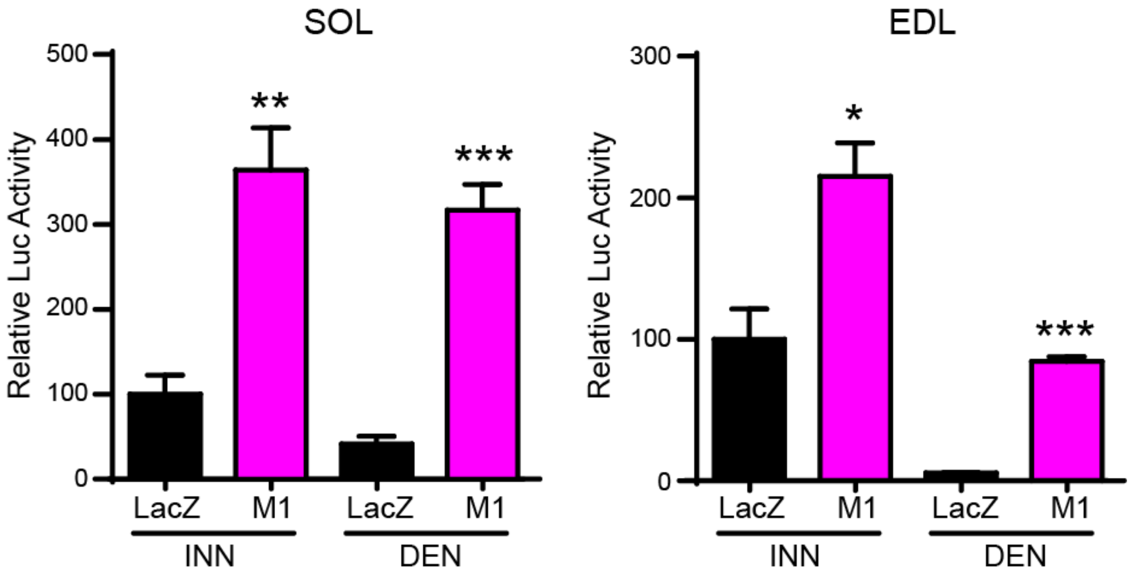

Supplementary Figure 7. The increase in MEF2 transcriptional activity induced by *Mrf4* knockdown (M1) is seen in both innervated and denervated SOL and EDL muscles. Muscles were transfected with a MEF2 reporter and analyzed after 7 days. Data are expressed as percent luciferase activity relative to control innervated muscles. Note that MEF2 reporter activity is reduced in denervated compared to innervated muscles especially in the EDL muscle. Data are presented as means±SEM from at least three independent experiments (n=4). Statistical analysis was performed using Student's two-tailed *t*-test (*p<0.05, **p<0.01, ***p<0.001).

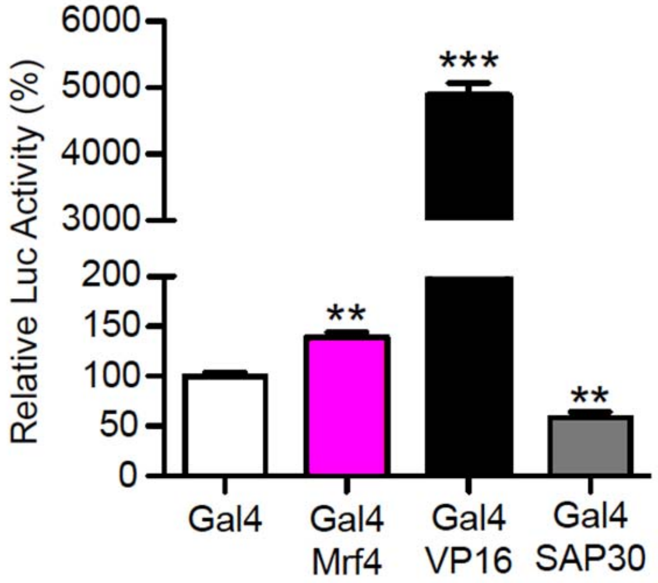

Supplementary Figure 8. **Effect of a GAL4-MRF4 chimera on GAL4 DNA-binding domain-luciferase reporter**. HEK 293 cells were co-transfected with a luciferase reporter, driven by five GAL4 binding sites upstream the TK minimal promoter, and either the basic GAL4 DNA-binding domain as a control or constructs encoding fusion proteins containing MRF4, VP16 or SAP30 cDNAs cloned in frame to the GAL4 DNA-binding domain. Data are expressed as percent luciferase activity relative to control and are presented as means±SEM from at least three independent experiments (n=4). Statistical analysis was performed using Student's two-tailed *t*-test (*p<0.05, **p<0.01, ***p<0.001).

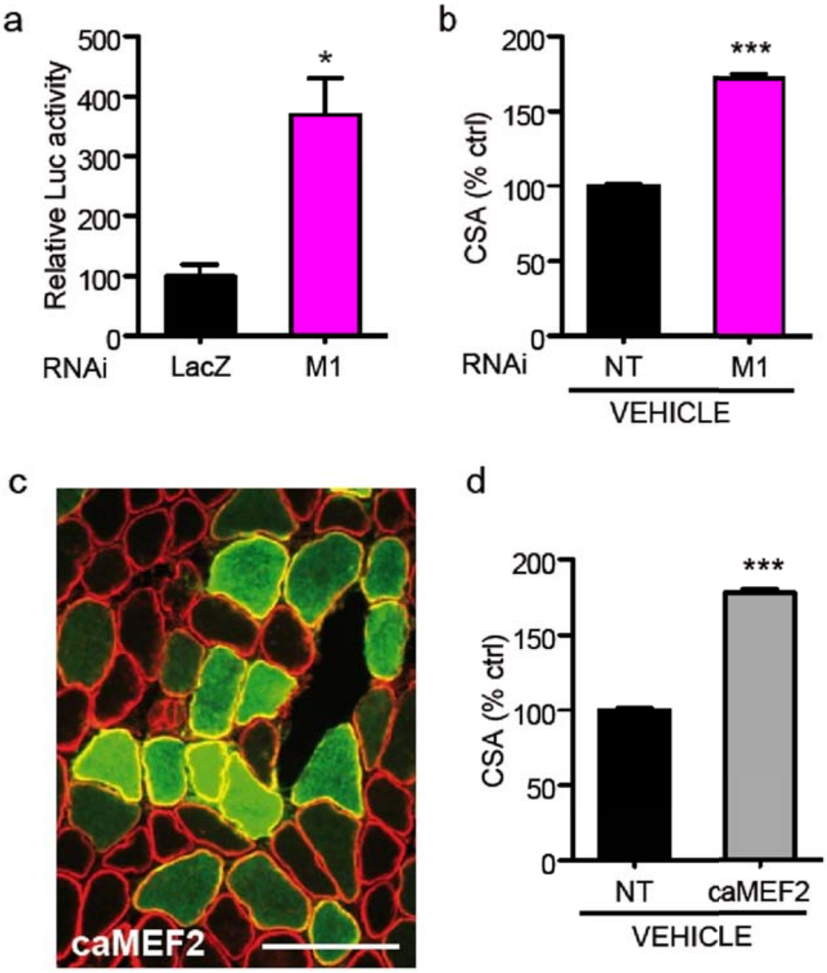

Supplementary Figure 9. Muscle hypertrophy is induced in mouse muscles by Mrf4 knockdown and constitutively active MEF2. (**a**) Increased MEF2 transcriptional activity induced by MRF4 RNAi compared to LacZ RNAi in SOL muscle from adult mouse. Data are expressed as percent luciferase activity relative to control muscles (n=4). **(b**) Increase in fiber size induced in adult mouse SOL muscle by Mrf4 knockdown (n=5). (**c**) Transverse section of adult mouse soleus muscle co-transfected with GFP and caMEF2 and stained for dystrophin and GFP. Scale bar 25 µm. (**d**) Increase in fiber size induced by caMEF2 (n=5). Data are presented as means±SEM, statistical analysis was performed using Student's two-tailed *t*-test (*p<0.05, **p<0.01, ***p<0.001).

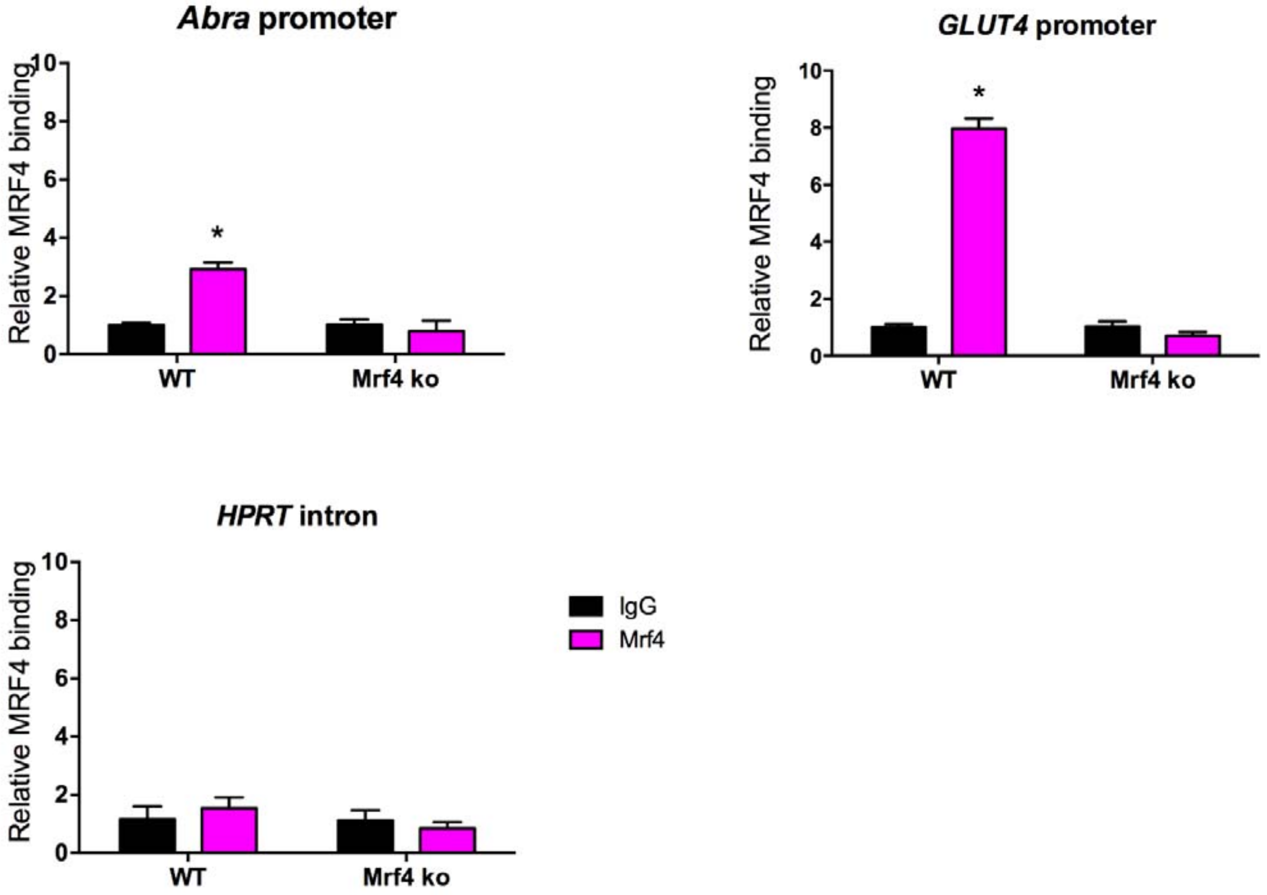

Supplementary Figure 10. MRF4 recruitment to MEF2 sites in the mouse Abra and Glut4 promoters. MRF4 binding was determined by ChIP-qPCR in denervated gastrocnemius muscle of adult wild type (WT) and *Mrf4* knockout (*Mrf4* KO) mice and expressed relative to IgG binding. An intronic region of Hprt was used as negative control. Data are presented as means±SEM from at least three independent experiments (n=3). Statistical analysis was performed using Student's two-tailed *t*-test (*p<0.05, **p<0.01, ***p<0.001).

Western blots shown in Fig. 1 and Supplementary Fig. 1

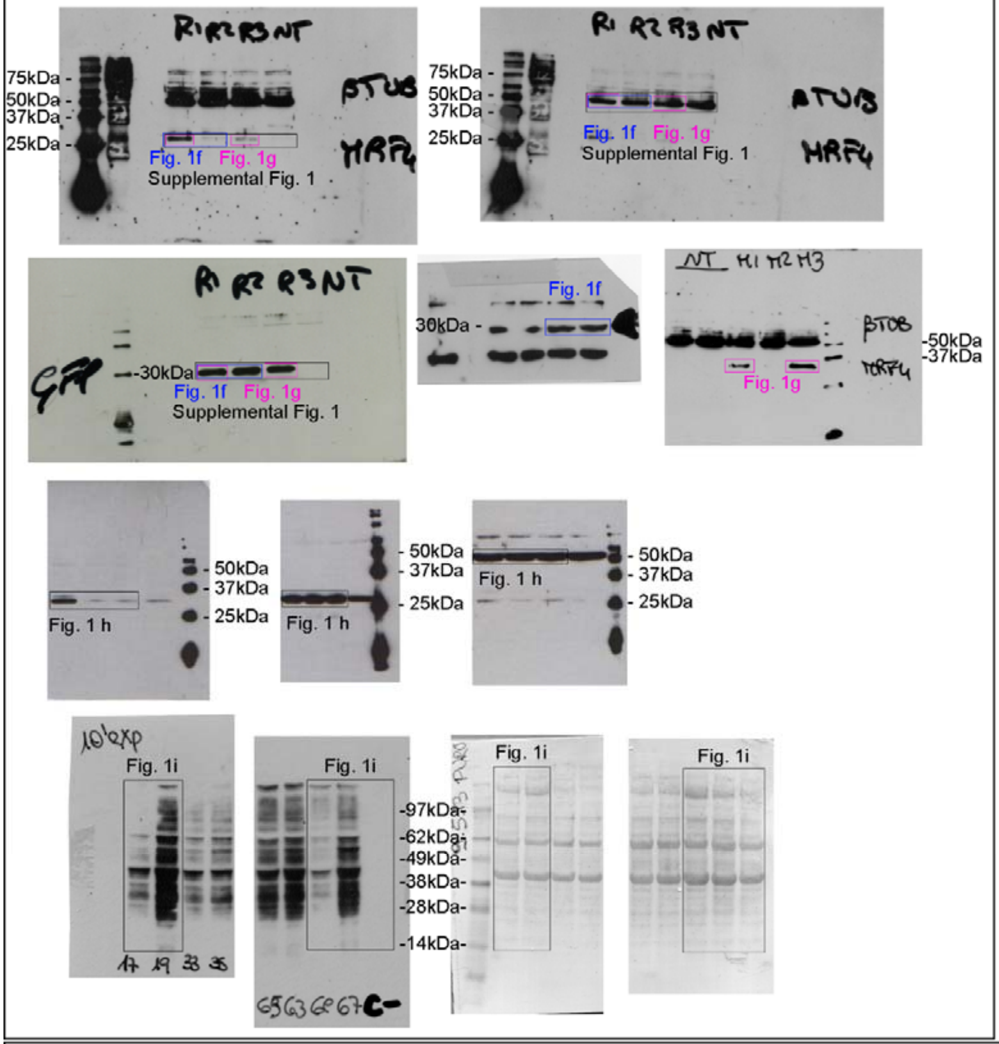

Western blots shown in Fig. 4

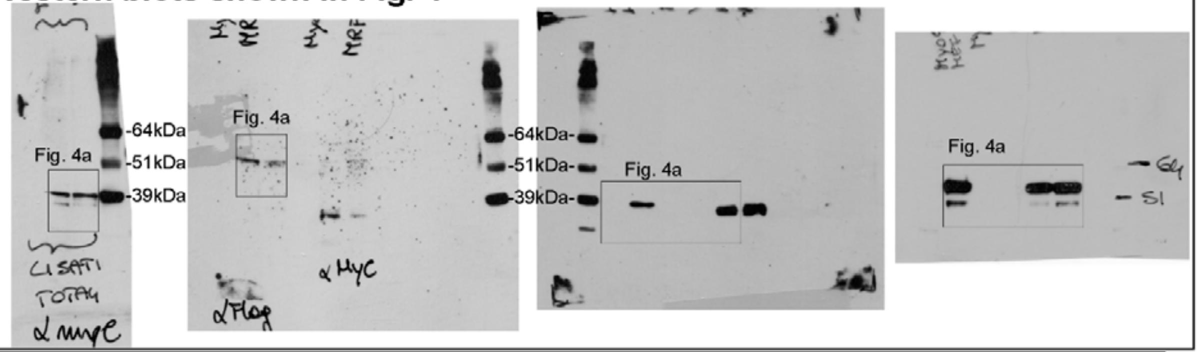

Western blots shown in Fig. 6

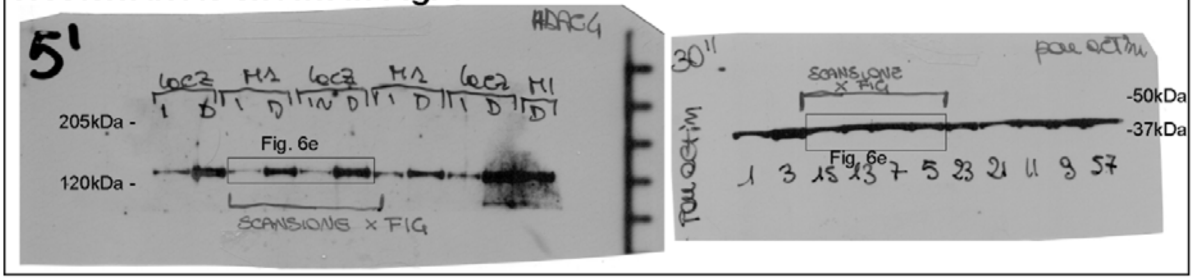

Supplementary Figure 11. Uncropped scans of Western Blots shown in Fig. 1, Fig. 4, Fig. 6 and Supplementary Fig. 1.

Supplementary Table 1. Selection of representative muscle genes upregulated in muscles transfected with MRF4 shRNAs compared to control LacZ shRNAs.

Gene	*Protein*	*Fold change*	*adj. p value*
Sarcolemma, T tubules			
Atp1b4	ATPase, (Na+)/K+ transporting, beta 4 polypeptide	19.24	8.63E-05
Scn4b	voltage-gated sodium channel, β4 subunit (Na_vβ4)	12.40	1.32E-04
Slc2a4	glucose transporter (GLUT4)	9.99	1.81E-05
Tfrc	transferrin receptor	8.80	6.86E-07
Chrnd	cholinergic receptor, nicotinic, delta	7.35	5.23E-06
Sspn	sarcospan	6.38	1.03E-05
Chrne	cholinergic receptor, nicotinic, epsilon	6.21	3.28E-05
Cacna2d1	calcium channel, voltage-dependent, alpha 2 delta 1 subunit	5.43	1.11E-05
Atp1b2	Na^+/K^+ATPase, beta 2 subunit	4.88	1.64E-05
Stac3	SH3 and cysteine rich domain 3	4.61	1.13E-03
Lamb3	laminin, beta 3	4.52	2.18E-04
Bin1	Bin1 (amphiphysin 2)	4.05	2.36E-05
Scn3b	sodium channel, voltage-gated, type III, beta	4.04	3.35E-06
Slc6a8	creatine transporter	4.03	1.48E-05
Vldlr	very low density lipoprotein receptor	3.44	2.80E-04
Cav3	caveolin 3	3.33	3.55E-04
Sarcoplasmic reticulum			
Dhrs7c	dehydrogenase/reductase (SDR family) member 7C (SRP-35)	10.46	3.96E-05
Casq1	calsequestrin 1 (fast)	9.38	1.03E-05
Tmem38a	transmembrane protein 38a (TRIC-A, trimeric intracellular cation channel A)	6.12	3.29E-04
Casq2	calsequestrin 2 (slow & cardiac)	5.53	6.53E-06
Srl	sarcalumenin	4.92	1.87E-05
Hrc	histidine rich calcium binding protein	4.44	1.82E-04
Rtn2	reticulon 2	4.40	1.87E-04
Ank1	ankyrin 1	3.45	1.27E-04
Contractile and cytoskeletal proteins (except myosins)			
Tnni3	troponin I type 3 (cardiac)	67.87	6.39E-06
Actn3	actinin alpha 3	21.39	4.75E-04
Mybpc2	myosin binding protein C, fast-type	18.16	5.92E-05
Myoz1	myozenin1 (calsarcin 2)	11.53	7.57E-06
Mylk2	myosin light chain kinase 2	10.71	2.82E-06

Lrrc39	leucine rich repeat containing 39 (myomasp)	9.72	4.86E-05
Ky	kyphoscoliosis peptidase	7.34	8.50E-06
Myot	myotilin	7.34	1.87E-05
Krt8	keratin 8	6.37	2.51E-03
Ldb3	LIM domain binding 3 (ZASP)	5.34	1.79E-04
Ankrd2	ankyrin repeat domain 2 (also nuclear)	5.46	1.97E-03
Synpo2	synaptopodin 2	4.87	1.05E-04
Tmod4	tropomodulin 4	4.78	1.34E-04
Ankrd23	ankyrin repeat domain 23 (DARP) (also nuclear)	4.35	1.73E-03
Pdlim3	PDZ and LIM domain 3	4.22	5.88E-04
Myom1	myomesin 1	4.20	3.56E-03
Tnnc2	troponin C type 2 (fast)	4.18	1.29E-04
Sgca	sarcoglycan, alpha	3.92	1.93E-04
Ttn	titin	3.58	3.67E-04
Mypn	myopalladin	3.41	3.07E-04
Sgcg	sarcoglycan, gamma	3.20	1.00E-03
Mybpc1	myosin binding protein C, slow-type	2.92	1.51E-04
Mitochondria			
Adck3	homolog of yeast COQ8 (Adck3)	13.85	2.82E-06
Bex1	brain expressed X-linked 1	8.46	5.50E-05
Me3	malic enzyme 3, NADP(+)-dependent, mitochondrial	7.10	4.38E-05
Reep1	receptor accessory protein 1	5.80	1.10E-04
Macrod1	MACRO domain containing 1	5.12	3.10E-04
Mtp18	18 kDa translocator protein (TSPO)	5.11	7.57E-06
Coq10a	coenzyme Q10 homolog A	3.87	1.18E-04
Acadm	acyl-CoA dehydrogenase, medium chain	3.77	1.48E-05
Ndufab1	NADH dehydrogenase (ubiquinone) 1, alpha/beta subcomplex, 1 (complex I subunit)	3.72	1.48E-05
Coq9	coenzyme Q9 homolog A	3.87	2.82E-04
Crat	carnitine O-acetyltransferase	3.44	2.68E-04
Hadhb	hydroxyacyl-CoA dehydrogenase/3-ketoacyl-CoA thiolase/ enoyl-CoA hydratase (trifunctional protein), beta subunit	3.40	4.81E-05
Aco2	aconitase 2, mitochondrial	3.38	7.22E-05
Idh3a	isocitrate dehydrogenase 3 (NAD+) alpha	3.38	1.52E-05
Pdk2	pyruvate dehydrogenase kinase, isozyme 2	3.35	9.04E-04
Cpt1b	carnitine palmitoyltransferase 1b, muscle	3.33	1.75E-03
Pdhx	pyruvate dehydrogenase complex, component X	3.26	9.15E-05
Acadvl	acyl-CoA dehydrogenase, very long chain	3.12	7.15E-04
Pdhb	pyruvate dehydrogenase (lipoamide) beta	2.94	7.73E-05
Hadh	hydroxyacyl-CoA dehydrogenase	2.92	7.27E-04
Cyc1	cytochrome c-1	2.69	1.42E-04
Glycolysis and glycogen metabolism			
Pgam2	phosphoglycerate mutase 2, muscle	10.53	2.82E-06
Ppp1r3c	protein phosphatase 1, regulatory (inhibitor) subunit 3C (PTG, protein targeting to glycogen)	11.85	1.95E-06
Ppp1r1a	protein phosphatase 1, regulatory (inhibitor) subunit 1A (I-1)	8.98	5.06E-05
Pygm	phosphorylase, glycogen, muscle	8.66	1.84E-04
Eno3	enolase 3, beta, muscle	7.54	1.87E-05

Pfkm	phosphofructokinase, muscle	6.31	2.80E-04
Gys1	glycogen synthase 1, muscle	6.28	1.61E-05
Agl	glycogen debranching enzyme (amylo- [illegible]-1,6-glucosidase, 4-[illegible]-glucanotransferase)	6.22	3.62E-05
Pgm1	phosphoglucomutase 1	5.96	1.52E-05
Pfkfb1	6-phosphofructo-2-kinase/fructose-2,6-biphosphatase 1	4.68	1.29E-05
Ldhb	lactate dehydrogenase B	4.14	1.51E-04
Gbe1	glucan (1,4-alpha-), branching enzyme 1	3.13	8.87E-04
Lipid metabolism (cytosolic proteins)			
Acsl6	acyl-CoA synthetase long-chain family member 6	7.05	6.15E-05
Pank1	pantothenate kinase 1	6.71	3.58E-06
Extl1	exostosin-like glycosyltransferase 1	6.14	2.34E-05
Me1	malic enzyme 1, NADP(+)-dependent, cytosolic	5.44	7.58E-05
Fabp3	fatty acid binding protein 3, muscle and heart	4.82	1.87E-05
Stress response			
Hspb6	Hsp20	6.93	3.58E-06
Mlf1	myeloid leukemia factor 1	6.81	2.18E-04
Hspb3	heat shock protein 3	5.51	4.99E-05
Gstm7	glutathione S-transferase, mu 7	5.21	8.10E-04
Hspb1	heat shock protein 1	2,93	1.16E-03
Hspb2	heat shock protein beta 2	2,75	1.63E-03
Miscellanea			
Pvalb	parvalbumin	53.22	3.99E-05
Ak1	adenylate kinase 1	24.45	6.86E-07
Abra	actin-binding Rho activating protein, STARS	13.56	3.58E-06
Ppargc1b	peroxisome proliferator-activated receptor gamma, coactivator 1 beta (PGC1-beta)	3.70	1.79E-03

Changes in muscles transfected with shRNAs against MRF4 compared to muscles transfected with shRNAs against LacZ. Pooled data from innervated and denervated muscles. Threshold: fold increase > 2 fold, p value < 0,001.

Supplementary Table 2. Sequences of the primers used for quantitative Real Time-PCR assays.

**Gene**	**Species**	**Sequence**
rCkm_FW	*Rat*	AAGGGTGGAGACGATCTGGA
rCkm_RV	*Rat*	TGTTGAGAGCTTCCACGGAC
rGlut4_FW	*Rat*	GCCTGCCCGAAAGAGTCTAA
rGlut4_RV	*Rat*	CAGTGGACGCTCTCTTTCCA
rHdac4_FW	*Rat*	ACTGGACAGCTCGCTGACCTCC
rHdac4_RV	*Rat*	ACACAGCCTACAGCCAGGCG
rMef2A_FW	*Rat*	TAAAATCGCACCTGGCTTGC
rMef2A_RV	*Rat*	GCTAATGTTGAGCTGGCTGC
rMef2C_FW	*Rat*	GCTTCAATACTGCCAGTGCG
rMef2C_RV	*Rat*	GTGGTACGGTCTCCCAACTG
rMef2D_FW	*Rat*	TCTCTGGCACTAAGGACCCC
rMef2D_RV	*Rat*	GAATGTCACCTGGCGGTTCC
rMyh6_FW	*Rat*	CAACCTGTCCAAGTTCCGCA
rMyh6_RV	*Rat*	CATCGTGCATTTTCTGCTTGGC
rMyod1_FW	*Rat*	CTGCGCAACGCCATCCGCTA
rMyod1_RV	*Rat*	GGGCCGCTGTAATCCATCATGCC
rMyog_FW	*Rat*	GCCGGTGGTACCCAGTGAATGC
rMyog_RV	*Rat*	AGGGAGTGCAGGTTGTGGGC
rRplp0_FW	*Rat*	CAATAAGGTGCCAGCTGCTGCC
rRplp0_RV	*Rat*	AGTGGTGATGCCCAAAGCTTGGAA
rMRF4_FW	*Rat*	CCCTTACAGCTACAAACCCAAG
rMRF4_RV	*Rat*	TGCTCCTCCTTCCTTAGCAG
rAbra_RV	*Rat*	GGACATGGAGGAGAGGCCTGAG
rAbra_RV	*Rat*	ACTGCTGCCACCTGCCTTTCA
rMyh7B_FW	*Rat*	GAGTGTGGAGCAGGTGGTATTT
rMyh7B_RV	*Rat*	GGACCCCAATGAAGAACTGA

Supplementary Table 3. Gene Set Enrichement Analysis (GSEA) showing the 10 top ranking gene sets (TRANSFAC) positively enriched in muscles transfected with shRNAs against MRF4 (M1 sequence).

Gene set	*Description*	*NES*	*FDR*
Mef2	Myocyte enhancer factor 2	2,41	<0,0001
Tbp	TATA box binding protein	2,25	0,0003
Tcf3	E12/E47	2,15	0,0003
Myod1	MyoD	2,01	0,0012
Sf1	Splicing factor 1	1,93	0,0022
Tcf4	Transcription fatcor 4	1,79	0,0091
Esrra	Estrogen-related receptor α	1,78	0,0097
Pou2f1	Oct1	1,77	0,0114
Myog	Myogenin	1,74	0,0127
Fos-Jun	AP-1	1,71	0,0167

Supplementary Table 4. List of sarcomeric myosins specifically expressed in skeletal muscle used to implement the GO biological set for GSEA analysis.

Sarcomeric myosins		
Gene	*Protein*	*Description*
Myh1	MyHC-2X	skeletal muscle, adult
Myh2	MyHC-2A	skeletal muscle, adult
Myh3	MyHC-emb	skeletal muscle, embryonic
Myh4	MyHC-2B	skeletal muscle, adult
Myh6	MyHC-α-cardiac	cardiac muscle, alpha
Myh7	MyHC-1/slow	cardiac muscle, beta, and slow skeletal muscle
Myh8	MyHC-neo	skeletal muscle, neonatal/perinatal
Myh15	MyHC-15	myosin heavy chain 15
Mylpf	MLC2f	myosin light chain phosphorylatable, fast skeletal muscle
Myl1	MLC1f	myosin light chain 1 fast
Myl2	MLC2s/v	myosin light chain 2 regulatory, cardiac, slow
Myl3	MLC1s/v	myosin light chain 3 alkali; ventricular, skeletal, slow
